# Supplementary material for: PAC-Mediated AKI Protection Is Critically Mediated but Does Not Exclusively Depend on Cell-Derived Microvesicles
Source: Int J Nephrol. 2021 Mar 9;2021:8864183. doi: 10.1155/2021/8864183 (PMC7969116; doi:10.1155/2021/8864183)
Supplement: Supplementary Materials — Supplementary Table S1: proteomic analyzes in detail. The table shows results for the following stimulatory conditions: Angi/Ctro (angiopoietin-2 in relation to controls), only in AngII (angiopoietin-2 only), BMP5/Ctr (bone morphogenetic protein-5 in relation to controls), AngII/Bmp5 (angiopoietin-2 in relation to bone morphogenetic protein-5). [file 8864183.f1.pdf]

Identified Proteins	Accession Number	Ratio Angi/Ctro
Isoform 2 of IQ calmodulin-binding motif-cor	IQCB1_MOUSE	0,00
Fermitin family homolog 3 OS=Mus musculu	URP2_MOUSE	0,00
GTP-binding protein OS=Mus musculus OX=1	Q61635_MOUSE	0,00
Nucleoprotein TPR OS=Mus musculus OX=10	TPR_MOUSE	0,00
Arachidonate 12-lipoxygenase, 12S-type OS=	LOX12_MOUSE	0,00
Isoform 2 of Trem-like transcript 1 protein O	TRML1_MOUSE	0,00
Protein kinase C OS=Mus musculus OX=1009	Q4VA93_MOUSE	0,00
Pyrin and HIN domain-containing protein 1 O	IFIX_MOUSE	0,00
EH domain-containing protein 4 OS=Mus mu	EHD4_MOUSE	0,00
Isoform 2 of Phosphatidylinositol 3,4,5-trisph	SHIP1_MOUSE	0,00
Calpain-2 catalytic subunit OS=Mus musculu	CAN2_MOUSE	0,00
SWI/SNF-related matrix-associated actin-de	SMCA5_MOUSE	0,00
Platelet factor 4 OS=Mus musculus OX=1009	PLF4_MOUSE	0,00
Ly6g6f protein OS=Mus musculus OX=10090	B2RXM6_MOUSE	0,00
Nuclear mitotic apparatus protein 1 OS=Mus	NUMA1_MOUSE	0,00
CCR4-NOT transcription complex subunit 9 O	CNOT9_MOUSE	0,00
EH domain-containing protein 1 OS=Mus mu	EHD1_MOUSE	0,00
V-type proton ATPase subunit B, brain isofor	VATB2_MOUSE	0,00
Immunoglobulin kappa variable 4-57 (Fragm	A0A0A6YYE7_MOUSE	0,00
Septin-7 OS=Mus musculus OX=10090 GN=S	SEPT7_MOUSE	0,00
Isoform 2 of CCR4-NOT transcription comple	CNOT1_MOUSE	0,00
Complement C4-B OS=Mus musculus OX=10	CO4B_MOUSE	0,00
Very-long-chain (3R)-3-hydroxyacyl-CoA dehy	HACD3_MOUSE	0,00
Isoform 2 of Protein PML OS=Mus musculus	PML_MOUSE	0,00
Bifunctional glutamate/proline--tRNA ligase	SYEP_MOUSE	0,00
Ribosome-binding protein 1 OS=Mus muscul	RRBP1_MOUSE	0,00
Bromodomain adjacent to zinc finger domai	A2AUY4_MOUSE	0,00
Copine-1 OS=Mus musculus OX=10090 GN=C	CPNE1_MOUSE	0,00
Tubulin beta-1 chain OS=Mus musculus OX=1	TBB1_MOUSE	0,00
Copine-3 OS=Mus musculus OX=10090 GN=C	CPNE3_MOUSE	0,00
T-complex protein 1 subunit epsilon OS=Mus	TCPE_MOUSE	0,00
Isoform 2 of Rootletin OS=Mus musculus OX	CROCC_MOUSE	0,00
Cytoplasmic dynein 1 heavy chain 1 OS=Mus	DYHC1_MOUSE	0,00
Isoform 2 of Tyrosine-protein kinase JAK3 OS	JAK3_MOUSE	0,00
Neutrophil collagenase OS=Mus musculus O	MMP8_MOUSE	0,00
RuvB-like 2 OS=Mus musculus OX=10090 GN	RUVB2_MOUSE	0,00
Branched-chain-amino-acid aminotransferas	BCAT2_MOUSE	0,00
Pleiotropic regulator 1 OS=Mus musculus OX	PLRG1_MOUSE	0,00
Clathrin interactor 1 OS=Mus musculus OX=1	Q5SUH6_MOUSE	0,00
REVERSED Serine/threonine-protein kinase U	ULK1_MOUSE	0,00
Thyroid hormone receptor-associated protein	TR150_MOUSE	0,00
Protein ABHD11 OS=Mus musculus OX=1009	ABHDB_MOUSE	0,00
REVERSED Titin OS=Mus musculus OX=1009	TITIN_MOUSE	0,00
Isoform HK1 of Hexokinase-1 OS=Mus muscu	HXK1_MOUSE	0,02
Coronin-1A OS=Mus musculus OX=10090 GN	COR1A_MOUSE	0,03
Coagulation factor V OS=Mus musculus OX=1	FA5_MOUSE	0,03
Peptidyl-prolyl cis-trans isomerase F, mitoch	PPIF_MOUSE	0,06

Endonuclease domain-containing 1 protein C	ENDD1_MOUSE	0,08
Marginal zone B- and B1-cell-specific protein	MZB1_MOUSE	0,09
Myosin-9 OS=Mus musculus OX=10090 GN=I	MYH9_MOUSE	0,10
Isoform 2 of Band 3 anion transport protein (	B3AT_MOUSE	0,10
Multimerin-1 OS=Mus musculus OX=10090 G	MMRN1_MOUSE	0,12
Isoform 2 of Tyrosine-protein phosphatase n	PTN6_MOUSE	0,14
Tropomodulin-3 OS=Mus musculus OX=1009	TMOD3_MOUSE	0,15
Isoform 2 of Heterogeneous nuclear ribonucl	HNRPK_MOUSE	0,15
Malectin OS=Mus musculus OX=10090 GN=M	MLEC_MOUSE	0,15
NADH dehydrogenase [ubiquinone] 1 alpha s	NDUA8_MOUSE	0,17
Lamin-B2 OS=Mus musculus OX=10090 GN=	LMNB2_MOUSE	0,18
Trafficking protein particle complex subunit 3	TPPC3_MOUSE	0,19
Isoform 2 of Programmed cell death protein	PDCD6_MOUSE	0,20
Coagulation factor XIII A chain OS=Mus musc	F13A_MOUSE	0,20
Isoform Delta of Lamina-associated polypept	LAP2B_MOUSE	0,20
Isoform 2 of MICOS complex subunit Mic60 (	MIC60_MOUSE	0,21
Pre-mRNA-processing-splicing factor 8 OS=M	PRP8_MOUSE	0,21
Polypyrimidine tract-binding protein 1 OS=M	PTBP1_MOUSE	0,21
Fibrinogen alpha chain OS=Mus musculus OX	FIBA_MOUSE	0,22
Isoform 2 of Calcium/calmodulin-dependent	KCC2D_MOUSE	0,23
Protein ERGIC-53 OS=Mus musculus OX=100	LMAN1_MOUSE	0,25
Isoform 2 of Heterogeneous nuclear ribonucl	HNRPU_MOUSE	0,25
Myosin regulatory light chain 12B OS=Mus m	ML12B_MOUSE	0,25
Protein NipSnap homolog 3B OS=Mus muscu	NPS3B_MOUSE	0,25
Isoform 2 of Galectin-9 OS=Mus musculus O	LEG9_MOUSE	0,25
Fibrinogen beta chain OS=Mus musculus OX=	FIBB_MOUSE	0,26
Endoplasmic reticulum resident protein 44 O	ERP44_MOUSE	0,27
Glia-derived nexin OS=Mus musculus OX=10	GDN_MOUSE	0,27
Talin-1 OS=Mus musculus OX=10090 GN=Tln	TLN1_MOUSE	0,28
Alpha-galactosidase A OS=Mus musculus OX	AGAL_MOUSE	0,29
Metalloproteinase inhibitor 3 OS=Mus musc	TIMP3_MOUSE	0,30
Fibrinogen gamma chain OS=Mus musculus	FIBG_MOUSE	0,31
Coatomer subunit beta' OS=Mus musculus O	COPB2_MOUSE	0,31
Isoform CNPI of 2',3'-cyclic-nucleotide 3'-pho	CN37_MOUSE	0,31
Enoyl-CoA delta isomerase 1, mitochondrial (	ECI1_MOUSE	0,31
von Willebrand factor OS=Mus musculus OX=	VWF_MOUSE	0,31
NADH dehydrogenase [ubiquinone] flavoprot	NDUV2_MOUSE	0,32
Component of Sp100-rs OS=Mus musculus O	CSPRS_MOUSE	0,33
Stress-70 protein, mitochondrial OS=Mus mu	GRP75_MOUSE	0,33
Trafficking protein particle complex subunit 4	TPPC4_MOUSE	0,33
Isoform 2 of Inter alpha-trypsin inhibitor, hea	ITIH4_MOUSE	0,33
Netrin-3 OS=Mus musculus OX=10090 GN=N	NET3_MOUSE	0,33
Isoform 2 of C-type lectin domain family 1 m	CLC1B_MOUSE	0,33
Nucleophosmin OS=Mus musculus OX=10090	NPM_MOUSE	0,33
Gelsolin OS=Mus musculus OX=10090 GN=G	GELS_MOUSE	0,35
Beta-2-microglobulin OS=Mus musculus OX=	B2MG_MOUSE	0,36
Acyl-coenzyme A thioesterase 2, mitochondr	ACOT2_MOUSE	0,36
Chitinase-like protein 3 OS=Mus musculus O	CHIL3_MOUSE	0,37

Lamin-B1 OS=Mus musculus OX=10090 GN=	LMNB1_MOUSE	0,37
Dolichyl-diphosphooligosaccharide--protein g	OST48_MOUSE	0,38
Transforming growth factor beta-1 proprotei	TGFB1_MOUSE	0,39
Filamin-A OS=Mus musculus OX=10090 GN=	FLNA_MOUSE	0,40
Isoform SERCA3A of Sarcoplasmic/endoplasr	AT2A3_MOUSE	0,40
Cleavage and polyadenylation specificity fact	CPSF5_MOUSE	0,41
26S proteasome non-ATPase regulatory subu	PSMD2_MOUSE	0,42
Fibroblast growth factor 2 OS=Mus musculus	FGF2_MOUSE	0,44
BTB/POZ domain-containing protein KCTD12	KCD12_MOUSE	0,44
Complement C3 OS=Mus musculus OX=1009	CO3_MOUSE	0,45
GrpE protein homolog 1, mitochondrial OS=M	GRPE1_MOUSE	0,46
Protein SEC13 homolog OS=Mus musculus O	SEC13_MOUSE	0,47
Isoform VEGF-1 of Vascular endothelial grow	VEGFA_MOUSE	0,47
Actin-related protein 2/3 complex subunit 5	ARPC5_MOUSE	0,47
Calpain small subunit 1 OS=Mus musculus O	CPNS1_MOUSE	0,50
Isoform 2 of Tenascin-N OS=Mus musculus C	TENN_MOUSE	0,50
Sorbitol dehydrogenase OS=Mus musculus O	DHSO_MOUSE	0,50

Identified Proteins	Accession Number	Ratio Angi/Ctro
Isoform 2 of F-actin-capping protein subu	CAPZB_MOUSE	2,00
Titin OS=Mus musculus OX=10090 GN=Tt	TITIN_MOUSE	2,00
Glucosamine-6-phosphate isomerase 1 O	GNPI1_MOUSE	2,00
Serine (Or cysteine) peptidase inhibitor, c	G3X8T9_MOUSE	2,00
Glutamate dehydrogenase 1, mitochondr	DHE3_MOUSE	2,00
Latent-transforming growth factor beta-b	LTBP2_MOUSE	2,00
40S ribosomal protein S18 OS=Mus musc	RS18_MOUSE	2,00
Cluster of Isoform 2 of Heterogeneous nu	HNRPF_MOUSE	2,00
Hypoxanthine-guanine phosphoribosyltran	HPRT_MOUSE	2,00
Ubiquitin-conjugating enzyme E2 L3 OS=M	UB2L3_MOUSE	2,00
Vesicle-associated membrane protein-as	VAPA_MOUSE	2,00
Disintegrin and metalloproteinase domai	ADAM9_MOUSE	2,00
Monocyte differentiation antigen CD14 O	CD14_MOUSE	2,00
Rho-related GTP-binding protein RhoC OS	RHOC_MOUSE	2,00
Transcobalamin-2 OS=Mus musculus OX=	TCO2_MOUSE	2,02
Carbonic anhydrase 1 OS=Mus musculus (	CAH1_MOUSE	2,03
Carbonic anhydrase 2 OS=Mus musculus (	CAH2_MOUSE	2,03
Metalloproteinase inhibitor 1 OS=Mus mu	TIMP1_MOUSE	2,07
Glutathione peroxidase 3 OS=Mus muscu	GPX3_MOUSE	2,09
40S ribosomal protein S4, X isoform OS=M	RS4X_MOUSE	2,13
Ferritin OS=Mus musculus OX=10090 GN=	Q9CPX4_MOUSE	2,14
H-2 class I histocompatibility antigen, K-K	G3UXW2_MOUSE	2,14
N(4)-(beta-N-acetylglucosaminyl)-L-aspa	ASPG_MOUSE	2,15
Peroxiredoxin-1 OS=Mus musculus OX=10	PRDX1_MOUSE	2,19
S-formylglutathione hydrolase OS=Mus m	H3BKH6_MOUSE	2,20
Delta(3,5)-Delta(2,4)-dienoyl-CoA isomer	ECH1_MOUSE	2,20
Ferritin heavy chain OS=Mus musculus OX	FRIH_MOUSE	2,21
Cluster of Fibronectin OS=Mus musculus (	A0A087WR50_MOUS	2,25
60S ribosomal protein L12 OS=Mus musc	RL12_MOUSE	2,25
Cofilin-1 OS=Mus musculus OX=10090 GN	COF1_MOUSE	2,28
Heme oxygenase 1 OS=Mus musculus OX	HMOX1_MOUSE	2,29
Collagen, type VI, alpha 3 OS=Mus muscu	E9PWQ3_MOUSE	2,32
Proteasome subunit beta type-6 OS=Mus	PSB6_MOUSE	2,33
Isoform 1 of Core histone macro-H2A.1 O	H2AY_MOUSE	2,33
Protein disulfide-isomerase A4 OS=Mus m	PDIA4_MOUSE	2,33
Peptidyl-prolyl cis-trans isomerase A OS=	PPIA_MOUSE	2,34
Syntenin-1 OS=Mus musculus OX=10090 (	SDCB1_MOUSE	2,38
Vinculin OS=Mus musculus OX=10090 GN	VINC_MOUSE	2,38
Glutaminyl-peptide cyclotransferase OS=	QPCT_MOUSE	2,40
Actin-related protein 2 OS=Mus musculus	ARP2_MOUSE	2,40
A disintegrin and metalloproteinase with	ATS1_MOUSE	2,40
Procollagen C-endopeptidase enhancer 1	PCOC1_MOUSE	2,42
Isoform 2 of Adenylate kinase 2, mitocho	KAD2_MOUSE	2,43
ATP synthase subunit beta, mitochondrial	ATPB_MOUSE	2,44
Thioredoxin domain-containing protein 5	TXND5_MOUSE	2,45
Platelet-activating factor acetylhydrolase	PAFA_MOUSE	2,46
Cluster of Thrombospondin-1 OS=Mus mu	Q80YQ1_MOUSE	2,46

Malate dehydrogenase, cytoplasmic OS=M	MDHC_MOUSE	2,50
60S ribosomal protein L17 OS=Mus musc	RL17_MOUSE	2,50
Nucleobindin-1 OS=Mus musculus OX=10	NUCB1_MOUSE	2,55
Osteopontin OS=Mus musculus OX=1009(	OSTP_MOUSE	2,59
Proteasome subunit alpha type-4 OS=Mu	PSA4_MOUSE	2,60
Collagen alpha-1(V) chain OS=Mus muscu	CO5A1_MOUSE	2,61
Proteasome subunit beta type-3 OS=Mus	PSB3_MOUSE	2,67
Collagen alpha-2(I) chain OS=Mus muscu	CO1A2_MOUSE	2,68
Collagen alpha-1(I) chain OS=Mus muscu	CO1A1_MOUSE	2,68
Cluster of Alpha-enolase OS=Mus muscul	ENOA_MOUSE	2,70
CD5 antigen-like OS=Mus musculus OX=1	CD5L_MOUSE	2,71
Alcohol dehydrogenase [NADP(+)] OS=Mu	AK1A1_MOUSE	2,71
Protein FAM3C OS=Mus musculus OX=10(	FAM3C_MOUSE	2,71
Destrin OS=Mus musculus OX=10090 GN=	DEST_MOUSE	2,71
Beta-glucuronidase OS=Mus musculus OX	BGLR_MOUSE	2,74
Dipeptidyl peptidase 2 OS=Mus musculus	DPP2_MOUSE	2,75
Phosphatidylinositol transfer protein alph	PIPNA_MOUSE	2,75
Granulins OS=Mus musculus OX=10090 G	GRN_MOUSE	2,76
Cluster of Elongation factor 1-alpha 1 OS=	EF1A1_MOUSE	2,80
Ras-related protein Rab-11B OS=Mus mu	RB11B_MOUSE	2,80
Cluster of Tropomyosin alpha-1 chain OS=	TPM1_MOUSE	2,85
Peptidyl-prolyl cis-trans isomerase C OS=	PPIC_MOUSE	2,87
Cluster of Glyceraldehyde-3-phosphate de	G3P_MOUSE	2,89
Proteasome subunit alpha type-5 OS=Mu	PSA5_MOUSE	2,90
Lysosomal alpha-mannosidase OS=Mus m	MA2B1_MOUSE	2,92
SPARC OS=Mus musculus OX=10090 GN=	SPRC_MOUSE	2,96
V-type proton ATPase subunit S1 OS=Mus	VAS1_MOUSE	3,00
Rab GDP dissociation inhibitor beta OS=M	GDIB_MOUSE	3,00
REVERSED Kinesin-like protein KIF26B OS	KI26B_MOUSE	3,00
Chloride intracellular channel protein 1 O	CLIC1_MOUSE	3,00
Ras suppressor protein 1 OS=Mus muscul	RSU1_MOUSE	3,00
Ear6 protein OS=Mus musculus OX=1009(	Q923L7_MOUSE	3,00
Protein FAM49B OS=Mus musculus OX=1	FA49B_MOUSE	3,00
Stromelysin-1 OS=Mus musculus OX=100	MMP3_MOUSE	3,00
Isoform 2 of Stabilin-1 OS=Mus musculus	STAB1_MOUSE	3,00
Isoform 2 of Vascular endothelial growth	VEGFC_MOUSE	3,00
Interleukin-18-binding protein OS=Mus m	I18BP_MOUSE	3,00
Isoform 2 of Hydroxyacylglutathione hydr	GLO2_MOUSE	3,00
Carbonyl reductase [NADPH] 1 OS=Mus m	CBR1_MOUSE	3,00
Eukaryotic translation initiation factor 3 s	EIF3K_MOUSE	3,00
Cluster of Tubulin beta-5 chain OS=Mus m	TBB5_MOUSE	3,05
14-3-3 protein zeta/delta OS=Mus muscu	1433Z_MOUSE	3,05
Palmitoyl-protein thioesterase 1 OS=Mus	PPT1_MOUSE	3,10
Cluster of 14-3-3 protein gamma OS=Mus	1433G_MOUSE	3,16
Aldose reductase OS=Mus musculus OX=	ALDR_MOUSE	3,17
Plasminogen activator inhibitor 1 OS=Mu	G5E899_MOUSE	3,19
Integrin beta-3 OS=Mus musculus OX=10(	ITB3_MOUSE	3,20
Growth factor receptor-bound protein 2 O	GRB2_MOUSE	3,25

Prolactin-2C2 OS=Mus musculus OX=1009	PR2C2_MOUSE	3,29
N-acetylglucosamine-6-sulfatase OS=Mu	GNS_MOUSE	3,29
Proprotein convertase subtilisin/kexin typ	PCSK9_MOUSE	3,33
Proteasome subunit beta type-1 OS=Mus	PSB1_MOUSE	3,38
Cluster of Extracellular matrix protein 1 C	F8WI14_MOUSE	3,39
Fructose-bisphosphate aldolase A OS=Mu	ALDOA_MOUSE	3,41
Dystroglycan OS=Mus musculus OX=1009	DAG1_MOUSE	3,45
Isoform PLEC-1I of Plectin OS=Mus muscu	PLEC_MOUSE	3,50
Isoform 2 of Periostin OS=Mus musculus	POSTN_MOUSE	3,50
Chloride intracellular channel protein 4 O	CLIC4_MOUSE	3,50
Acid sphingomyelinase-like phosphodiest	ASM3A_MOUSE	3,50
Transgelin-2 OS=Mus musculus OX=1009	TAGL2_MOUSE	3,51
Follistatin-related protein 1 OS=Mus mus	FSTL1_MOUSE	3,61
Nidogen-1 OS=Mus musculus OX=10090	NID1_MOUSE	3,64
Lactoylglutathione lyase OS=Mus musculu	LGUL_MOUSE	3,67
Protein-lysine 6-oxidase OS=Mus musculu	LYOX_MOUSE	3,67
Carbonyl reductase [NADPH] 3 OS=Mus m	CBR3_MOUSE	3,67
Vimentin OS=Mus musculus OX=10090 G	VIME_MOUSE	3,70
Acid ceramidase OS=Mus musculus OX=1	ASAH1_MOUSE	3,73
Isoform II of Gamma-glutamyl hydrolase	GGH_MOUSE	3,75
Putative phospholipase B-like 2 OS=Mus	PLBL2_MOUSE	3,78
Translationally-controlled tumor protein	TCTP_MOUSE	3,88
40S ribosomal protein SA OS=Mus muscu	RSSA_MOUSE	3,90
Serotransferrin OS=Mus musculus OX=10	TRFE_MOUSE	3,97
Carboxypeptidase E OS=Mus musculus OX	CBPE_MOUSE	4,00
Ribonuclease T2-A OS=Mus musculus OX	RNT2A_MOUSE	4,00
Proteasome subunit alpha type-7 OS=Mu	PSA7_MOUSE	4,00
Protein/nucleic acid deglycase DJ-1 OS=M	PARK7_MOUSE	4,00
Protein CTLA-2-alpha OS=Mus musculus	CTL2A_MOUSE	4,00
Angiopoietin-related protein 2 OS=Mus m	ANGL2_MOUSE	4,00
Pro-cathepsin H OS=Mus musculus OX=1	CATH_MOUSE	4,00
60S ribosomal protein L6 OS=Mus muscu	RL6_MOUSE	4,00
Desmin OS=Mus musculus OX=10090 GN	DESM_MOUSE	4,00
COP9 signalosome complex subunit 8 OS	CSN8_MOUSE	4,00
[Protein ADP-ribosylarginine] hydrolase O	ADPRH_MOUSE	4,00
Transcriptional activator protein Pur-alph	PURA_MOUSE	4,00
14-3-3 protein eta OS=Mus musculus OX=	1433F_MOUSE	4,04
Cluster of Insulin-like growth factor-bindi	IBP7_MOUSE	4,05
Glutathione S-transferase P 1 OS=Mus m	GSTP1_MOUSE	4,14
Tubulointerstitial nephritis antigen-like O	TINAL_MOUSE	4,17
Biglycan OS=Mus musculus OX=10090 GN	PGS1_MOUSE	4,18
Annexin A1 OS=Mus musculus OX=10090	ANXA1_MOUSE	4,21
Leukocyte elastase inhibitor A OS=Mus m	ILEUA_MOUSE	4,25
Cofilin-2 OS=Mus musculus OX=10090 GN	COF2_MOUSE	4,25
14-3-3 protein epsilon OS=Mus musculus	1433E_MOUSE	4,26
Transgelin OS=Mus musculus OX=10090	TAGL_MOUSE	4,29
Cluster of Heat shock protein HSP 90-beta	HS90B_MOUSE	4,33
6-phosphogluconolactonase OS=Mus mus	6PGL_MOUSE	4,33

Secreted frizzled-related protein 2 OS=M	SFRP2_MOUSE	4,33
N-acetylgalactosamine-6-sulfatase OS=M	GALNS_MOUSE	4,33
Isoform 2 of Neutral alpha-glucosidase A	GANAB_MOUSE	4,33
Rho GDP-dissociation inhibitor 1 OS=Mus	GDIR1_MOUSE	4,43
Bisphosphoglycerate mutase OS=Mus mu	PMGE_MOUSE	4,43
C-C motif chemokine 2 OS=Mus musculus	CCL2_MOUSE	4,50
Glucose-6-phosphate isomerase OS=Mus	G6PI_MOUSE	4,50
Biliverdin reductase A OS=Mus musculus	BIEA_MOUSE	4,50
Proteasome subunit beta type-4 OS=Mus	PSB4_MOUSE	4,50
Cathepsin O OS=Mus musculus OX=10090	CATO_MOUSE	4,50
Moesin OS=Mus musculus OX=10090 GN	MOES_MOUSE	4,57
Alpha-N-acetylglucosaminidase OS=Mus	O88325_MOUSE	4,67
Insulin-like growth factor-binding protein	IBP4_MOUSE	4,67
Procollagen-lysine,2-oxoglutarate 5-dioxy	PLOD1_MOUSE	4,71
Isoform 2 of Fibulin-2 OS=Mus musculus	FBLN2_MOUSE	4,80
Cluster of Pyruvate kinase PKM OS=Mus r	KPYM_MOUSE	4,86
Isoform 2 of 14-3-3 protein theta OS=Mu	1433T_MOUSE	5,00
Integrin alpha-5 OS=Mus musculus OX=1	ITA5_MOUSE	5,00
Cluster of Ubiquitin-conjugating enzyme	UB2V1_MOUSE	5,00
Glutathione S-transferase A4 OS=Mus mu	GSTA4_MOUSE	5,00
Lysyl oxidase homolog 2 OS=Mus musculu	LOXL2_MOUSE	5,00
Isoform Alpha-6X1A of Integrin alpha-6 O	ITA6_MOUSE	5,00
Cluster of Caldesmon 1 OS=Mus musculu	E9QA16_MOUSE	5,00
Serpin H1 OS=Mus musculus OX=10090 G	SERPH_MOUSE	5,05
Isoform 2 of Sorbin and SH3 domain-cont	SRBS2_MOUSE	5,25
Phosphoglycerate kinase 1 OS=Mus musc	PGK1_MOUSE	5,27
Peroxiredoxin-6 OS=Mus musculus OX=10	PRDX6_MOUSE	5,32
Galectin-3-binding protein OS=Mus musc	LG3BP_MOUSE	5,33
Phosphatidylethanolamine-binding protei	PEBP1_MOUSE	5,33
Actin-related protein 2/3 complex subuni	ARC1B_MOUSE	5,33
Cluster of Collagen alpha-1(XVIII) chain O	COIA1_MOUSE	5,47
Ribosomal protein OS=Mus musculus OX=	Q5XJF6_MOUSE	5,50
Follistatin-related protein 3 OS=Mus mus	FSTL3_MOUSE	5,50
Proteasome subunit alpha type-1 OS=Mu	PSA1_MOUSE	5,83
Cluster of Alpha-actinin-4 OS=Mus muscu	ACTN4_MOUSE	5,89
Proteasome subunit beta type-5 OS=Mus	PSB5_MOUSE	6,00
Integrin beta-1 OS=Mus musculus OX=10	ITB1_MOUSE	6,00
Elongation factor 1-beta OS=Mus muscul	EF1B_MOUSE	6,00
Phospholipase B-like 1 OS=Mus musculus	PLBL1_MOUSE	6,00
Isoform 2 of Septin-11 OS=Mus musculus	SEP11_MOUSE	6,00
Tripeptidyl-peptidase 1 OS=Mus musculu	TPP1_MOUSE	6,22
Serpin B6 OS=Mus musculus OX=10090 G	SPB6_MOUSE	6,29
Elongation factor 1-gamma OS=Mus mus	EF1G_MOUSE	6,29
6-phosphogluconate dehydrogenase, deca	6PGD_MOUSE	6,40
Phosphatidylinositol transfer protein beta	PIPNB_MOUSE	6,50
Amyloid-beta A4 protein OS=Mus muscul	A4_MOUSE	6,60
Annexin A5 OS=Mus musculus OX=10090	ANXA5_MOUSE	6,60
Synaptic vesicle membrane protein VAT-1	VAT1_MOUSE	6,88

Annexin A2 OS=Mus musculus OX=10090	ANXA2_MOUSE	6,88
Proteasome subunit alpha type-6 OS=Mu	PSA6_MOUSE	7,00
Proteasome subunit beta type-2 OS=Mus	PSB2_MOUSE	7,00
Beta-1,3-N-acetylglucosaminyltransferas	LFNG_MOUSE	7,00
Fibulin-1 OS=Mus musculus OX=10090 GI	FBLN1_MOUSE	7,00
72 kDa type IV collagenase OS=Mus musc	MMP2_MOUSE	7,20
Phospholipase D3 OS=Mus musculus OX=	PLD3_MOUSE	7,33
Retinoid-inducible serine carboxypeptidas	RISC_MOUSE	7,33
Transketolase OS=Mus musculus OX=100	TKT_MOUSE	7,60
Neuronal pentraxin-1 OS=Mus musculus (	NPTX1_MOUSE	8,00
Endothelial protein C receptor OS=Mus m	EPCR_MOUSE	8,00
Cadherin-11 OS=Mus musculus OX=10090	CAD11_MOUSE	8,00
Fibrillin-1 OS=Mus musculus OX=10090 G	FBN1_MOUSE	8,50
Clusterin OS=Mus musculus OX=10090 GI	CLUS_MOUSE	8,61
Connective tissue growth factor OS=Mus	CTGF_MOUSE	8,89
Inactive tyrosine-protein kinase 7 OS=Mu	PTK7_MOUSE	9,00
C-X-C motif chemokine 16 OS=Mus muscu	CXL16_MOUSE	9,00
Cluster of Heat shock 70 kDa protein 4 OS	HSP74_MOUSE	10,00
Twinfilin-1 OS=Mus musculus OX=10090	TWF1_MOUSE	10,00
Mammalian ependymin-related protein 1	EPDR1_MOUSE	10,00
Nuclease-sensitive element-binding prote	YBOX1_MOUSE	10,00
AHNAK nucleoprotein (desmoyokin) OS=M	E9Q616_MOUSE	10,00
Isoform 2 of Heterogeneous nuclear ribor	HNRPD_MOUSE	10,00
Isoform 2 of Tenascin OS=Mus musculus	TENA_MOUSE	10,20
Protein CYR61 OS=Mus musculus OX=100	CYR61_MOUSE	10,20
Collagen alpha-2(IV) chain OS=Mus musc	CO4A2_MOUSE	10,30
Testin-2 OS=Mus musculus OX=10090 PE	TEST2_MOUSE	10,50
Glutathione S-transferase omega-1 OS=M	GSTO1_MOUSE	10,50
Basement membrane-specific heparan su	E9PZ16_MOUSE	10,52
Poly(rC)-binding protein 1 OS=Mus muscu	PCBP1_MOUSE	10,67
Isocitrate dehydrogenase [NADP] cytoplas	IDHC_MOUSE	11,00
40S ribosomal protein S3 OS=Mus muscu	RS3_MOUSE	11,00
Carboxypeptidase Q OS=Mus musculus O	CBPQ_MOUSE	11,00
Isoform 2 of Proliferation-associated prot	PA2G4_MOUSE	11,00
Bone morphogenetic protein 1 OS=Mus m	BMP1_MOUSE	11,25
Cluster of Glutathione S-transferase Mu 1	GSTM1_MOUSE	11,75
WD repeat-containing protein 1 OS=Mus	WDR1_MOUSE	12,00
Isoform LAMP-2B of Lysosome-associate	LAMP2_MOUSE	12,00
Dihydropteridine reductase OS=Mus musc	DHPR_MOUSE	12,00
PDZ and LIM domain protein 5 OS=Mus m	PDLI5_MOUSE	12,50
Isoform 2 of Disintegrin and metalloprote	ADA15_MOUSE	13,00
Prolow-density lipoprotein receptor-relate	LRP1_MOUSE	14,00
60S ribosomal protein L5 OS=Mus muscu	RL5_MOUSE	14,00
Isoform Mt-VDAC1 of Voltage-dependent	VDAC1_MOUSE	14,00
Mannosyl-oligosaccharide 1,2-alpha-man	MA1A1_MOUSE	14,00
Phospholipid transfer protein OS=Mus mu	PLTP_MOUSE	14,50
Cysteine and glycine-rich protein 1 OS=M	CSRP1_MOUSE	14,50
Ras-related C3 botulinum toxin substrate	RAC1_MOUSE	14,50

Aspartate aminotransferase, cytoplasmic	AATC_MOUSE	15,00
ATP synthase subunit O, mitochondrial O	ATPO_MOUSE	15,00
Proteasome subunit beta type-8 OS=Mus	PSB8_MOUSE	16,00
Collagen alpha-1(IV) chain OS=Mus musc	CO4A1_MOUSE	16,25
Angiopoietin-1 OS=Mus musculus OX=10	ANGP1_MOUSE	17,47
Receptor of activated protein C kinase 1	RACK1_MOUSE	17,50
Ribonuclease inhibitor OS=Mus musculus	RINI_MOUSE	18,00
Thioredoxin-like protein 1 OS=Mus muscu	TXNL1_MOUSE	18,00
Collagen alpha-1(VI) chain OS=Mus musc	CO6A1_MOUSE	18,50
Insulin-like growth factor-binding protein	IBP3_MOUSE	19,00
Elongation factor 2 OS=Mus musculus OX	EF2_MOUSE	19,33
Dihydropyrimidinase-related protein 2 OS	DPYL2_MOUSE	20,00
Epididymis-specific alpha-mannosidase O	MA2B2_MOUSE	21,00
Isoform 2 of Sulfhydryl oxidase 1 OS=Mus	QSOX1_MOUSE	21,33
Adipocyte enhancer-binding protein 1 OS=	AEBP1_MOUSE	28,50
Collagen alpha-2(V) chain OS=Mus muscu	CO5A2_MOUSE	30,00
Peroxidasin homolog OS=Mus musculus O	PXDN_MOUSE	59,50
Fascin OS=Mus musculus OX=10090 GN=	FSCN1_MOUSE	67,00
Cadherin-5 OS=Mus musculus OX=10090	CADH5_MOUSE	141,00
Angiopoietin-2 OS=Mus musculus OX=10	ANGP2_MOUSE	only in AngII
Filamin-B OS=Mus musculus OX=10090 G	FLNB_MOUSE	only in AngII
Endothelial cell-specific molecule 1 OS=M	ESM1_MOUSE	only in AngII
Myosin light chain 3 OS=Mus musculus O	MYL3_MOUSE	only in AngII
Vascular cell adhesion protein 1 OS=Mus	VCAM1_MOUSE	only in AngII
Isoform 2 of Cell surface glycoprotein MU	MUC18_MOUSE	only in AngII
Beta-galactosidase OS=Mus musculus OX	BGAL_MOUSE	only in AngII
Myosin regulatory light chain 2, ventricula	MLRV_MOUSE	only in AngII
Isoform 2 of Lactadherin OS=Mus muscul	MFGM_MOUSE	only in AngII
Complement component C1q receptor OS	C1QR1_MOUSE	only in AngII
Transitional endoplasmic reticulum ATPa	TERA_MOUSE	only in AngII
60S acidic ribosomal protein P0 OS=Mus	RLA0_MOUSE	only in AngII
Coiled-coil domain-containing protein 80	CCD80_MOUSE	only in AngII
Tyrosine-protein kinase receptor Tie-1 OS	TIE1_MOUSE	only in AngII
Dihydropyrimidinase-related protein 3 OS	E9PWE8_MOUSE	only in AngII
Fibulin-5 OS=Mus musculus OX=10090 GI	FBLN5_MOUSE	only in AngII
Isoform 2 of Calsyntenin-1 OS=Mus musc	CSTN1_MOUSE	only in AngII
Phosphoserine aminotransferase OS=Mus	SERC_MOUSE	only in AngII
Semaphorin-7A OS=Mus musculus OX=10	SEM7A_MOUSE	only in AngII
Plasmalemma vesicle-associated protein	PLVAP_MOUSE	only in AngII
Complement C1s-A subcomponent OS=M	CS1A_MOUSE	only in AngII
Calcium-activated chloride channel regula	CA3A1_MOUSE	only in AngII
Isoform 2 of Platelet endothelial cell adhe	PECA1_MOUSE	only in AngII
Vitamin K-dependent protein S OS=Mus r	PROS_MOUSE	only in AngII
40S ribosomal protein S5 (Fragment) OS=	D3YYM6_MOUSE	only in AngII
Cluster of H-2 class I histocompatibility a	HA11_MOUSE	only in AngII
60S ribosomal protein L7 OS=Mus muscu	RL7_MOUSE	only in AngII
40S ribosomal protein S3a OS=Mus musc	RS3A_MOUSE	only in AngII
Nidogen-2 OS=Mus musculus OX=10090	NID2_MOUSE	only in AngII

Aconitate hydratase, mitochondrial OS=M	ACON_MOUSE	only in AngII
Isoform A3B of Troponin T, cardiac muscl	TNNT2_MOUSE	only in AngII
Aldose reductase-related protein 2 OS=M	ALD2_MOUSE	only in AngII
Laminin subunit alpha-4 OS=Mus musculu	LAMA4_MOUSE	only in AngII
40S ribosomal protein S2 OS=Mus muscu	RS2_MOUSE	only in AngII
Isoform 3 of Programmed cell death 6-int	PDC6I_MOUSE	only in AngII
NK13 OS=Mus musculus OX=10090 GN=S	O08804_MOUSE	only in AngII
Glypican-4 OS=Mus musculus OX=10090 (	GPC4_MOUSE	only in AngII
Myoglobin OS=Mus musculus OX=10090 (	MYG_MOUSE	only in AngII
Arylsulfatase A OS=Mus musculus OX=10	ARSA_MOUSE	only in AngII
Proteasome subunit beta type-7 OS=Mus	PSB7_MOUSE	only in AngII
Troponin I, cardiac muscle OS=Mus musc	TNNI3_MOUSE	only in AngII
Macrophage colony-stimulating factor 1 (	CSF1_MOUSE	only in AngII
Collagen alpha-1(XV) chain OS=Mus musc	COFA1_MOUSE	only in AngII
Lysyl oxidase homolog 3 OS=Mus musculu	LOXL3_MOUSE	only in AngII
Decorin OS=Mus musculus OX=10090 GN	PGS2_MOUSE	only in AngII
Spermidine synthase OS=Mus musculus (	SPEE_MOUSE	only in AngII
Laminin subunit gamma-1 OS=Mus musc	F8VQJ3_MOUSE	only in AngII
Ras-related protein Rab-7a OS=Mus mus	RAB7A_MOUSE	only in AngII
Adenosylhomocysteinase OS=Mus muscu	SAHH_MOUSE	only in AngII
Coronin-1C OS=Mus musculus OX=10090	COR1C_MOUSE	only in AngII
Trifunctional enzyme subunit alpha, mito	ECHA_MOUSE	only in AngII
Low-density lipoprotein receptor OS=Mus	LDLR_MOUSE	only in AngII
GTP-binding nuclear protein Ran OS=Mus	RAN_MOUSE	only in AngII
Selenoprotein P OS=Mus musculus OX=10	SEPP1_MOUSE	only in AngII
Isoform 2 of Heterogeneous nuclear ribor	ROA2_MOUSE	only in AngII
Calumenin OS=Mus musculus OX=10090 (	CALU_MOUSE	only in AngII
UMP-CMP kinase OS=Mus musculus OX=1	KCY_MOUSE	only in AngII
Heterogeneous nuclear ribonucleoprotein	ROAA_MOUSE	only in AngII
40S ribosomal protein S8 OS=Mus muscu	RS8_MOUSE	only in AngII
Electron transfer flavoprotein subunit alp	ETFA_MOUSE	only in AngII
Electron transfer flavoprotein subunit bet	ETFB_MOUSE	only in AngII
Plasma protease C1 inhibitor OS=Mus mu	IC1_MOUSE	only in AngII
Laminin subunit beta-1 OS=Mus musculus	LAMB1_MOUSE	only in AngII
Lysyl oxidase homolog 1 OS=Mus musculu	LOXL1_MOUSE	only in AngII
Cluster of Myosin-binding protein C, cardi	E9Q9T8_MOUSE	only in AngII
Creatine kinase M-type OS=Mus musculus	KCRM_MOUSE	only in AngII
Sepiapterin reductase OS=Mus musculus	SPRE_MOUSE	only in AngII
Beta-mannosidase OS=Mus musculus OX	A0A0R4J092_MOUSE	only in AngII
Eukaryotic initiation factor 4A-I OS=Mus r	IF4A1_MOUSE	only in AngII
60S ribosomal protein L18 OS=Mus musc	RL18_MOUSE	only in AngII
T-complex protein 1 subunit eta OS=Mus	TCPH_MOUSE	only in AngII
3-ketoacyl-CoA thiolase, mitochondrial OS	THIM_MOUSE	only in AngII
Angiotensin-converting enzyme OS=Mus	ACE_MOUSE	only in AngII
Isoform Beta of Tissue factor pathway inh	TFPI1_MOUSE	only in AngII
Ceroid-lipofuscinosis neuronal protein 5 h	CLN5_MOUSE	only in AngII
Cytochrome c oxidase subunit 5A, mitoch	COX5A_MOUSE	only in AngII
Microtubule-associated protein RP/EB fa	MARE1_MOUSE	only in AngII

Isoform Cytoplasmic+peroxisomal of Perc	PRDX5_MOUSE	only in AngII
Peptidyl-prolyl cis-trans isomerase FKBP1	FKB10_MOUSE	only in AngII
Serine protease HTRA1 OS=Mus musculu	HTRA1_MOUSE	only in AngII
NAD(P) transhydrogenase, mitochondrial	NNTM_MOUSE	only in AngII
Proteasome activator complex subunit 1 (	PSME1_MOUSE	only in AngII
60S ribosomal protein L14 OS=Mus musc	RL14_MOUSE	only in AngII
60S ribosomal protein L7a OS=Mus musc	RL7A_MOUSE	only in AngII
Isoform 2 of AP-2 complex subunit beta C	AP2B1_MOUSE	only in AngII
Ephrin-A1 OS=Mus musculus OX=10090 G	EFNA1_MOUSE	only in AngII
Macrophage colony-stimulating factor 1 r	CSF1R_MOUSE	only in AngII
60S ribosomal protein L18a OS=Mus mus	RL18A_MOUSE	only in AngII
Ubiquitin carboxyl-terminal hydrolase iso	UCHL1_MOUSE	only in AngII
Zyxin OS=Mus musculus OX=10090 GN=Z	ZYX_MOUSE	only in AngII
Isoform A0 of Neuropilin-2 OS=Mus musc	NRP2_MOUSE	only in AngII
Isoform 2 of Cytosol aminopeptidase OS=	AMPL_MOUSE	only in AngII
Isoform 2 of Vacuolar protein sorting-ass	VPS29_MOUSE	only in AngII
60S ribosomal protein L9 OS=Mus muscu	RL9_MOUSE	only in AngII
ATP synthase subunit delta, mitochondria	ATPD_MOUSE	only in AngII
Glutathione peroxidase 1 OS=Mus muscu	GPX1_MOUSE	only in AngII
Inhibin beta A chain OS=Mus musculus O	INHBA_MOUSE	only in AngII
Osteoclast-stimulating factor 1 OS=Mus r	OSTF1_MOUSE	only in AngII
60S ribosomal protein L13 OS=Mus musc	RL13_MOUSE	only in AngII
Acetyl-CoA acetyltransferase, mitochondr	THIL_MOUSE	only in AngII
Ubiquitin-conjugating enzyme E2 K OS=M	UBE2K_MOUSE	only in AngII
Synaptobrevin homolog YKT6 OS=Mus mu	YKT6_MOUSE	only in AngII
Lymphocyte antigen 86 OS=Mus musculu	LY86_MOUSE	only in AngII
Tyrosine-protein kinase Mer OS=Mus mus	MERTK_MOUSE	only in AngII
Olfactomedin-like protein 2B OS=Mus mu	OLM2B_MOUSE	only in AngII
40S ribosomal protein S7 OS=Mus muscu	RS7_MOUSE	only in AngII
T-complex protein 1 subunit zeta OS=Mus	TCPZ_MOUSE	only in AngII
Ubiquitin-like modifier-activating enzyme	UBA1_MOUSE	only in AngII
Isoform 2 of Matrilin-2 OS=Mus musculus	MATN2_MOUSE	only in AngII
Isoform 2 of Bone morphogenetic protein	BMP5_MOUSE	only in AngII
Major prion protein OS=Mus musculus OX	PRIO_MOUSE	only in AngII
Vascular endothelial growth factor recept	VGFR1_MOUSE	only in AngII
Cluster of Isoform 2 of Microtubule-assoc	MAP4_MOUSE	only in AngII
Fatty acid synthase OS=Mus musculus OX	FAS_MOUSE	only in AngII
Farnesyl pyrophosphate synthase OS=Mus	FPPS_MOUSE	only in AngII
Isopentenyl-diphosphate Delta-isomerase	IDI1_MOUSE	only in AngII
Integrin beta-2 OS=Mus musculus OX=10	ITB2_MOUSE	only in AngII
Platelet-activating factor acetylhydrolase	PA1B2_MOUSE	only in AngII
Proteasome activator complex subunit 2 (	PSME2_MOUSE	only in AngII
Cytochrome b-c1 complex subunit 2, mito	QCR2_MOUSE	only in AngII
Isoform 2 of 4F2 cell-surface antigen hea	4F2_MOUSE	only in AngII
Isoform 2 of Thioredoxin reductase 1, cyt	TRXR1_MOUSE	only in AngII
26S proteasome non-ATPase regulatory s	PSD13_MOUSE	only in AngII
Isoform 2 of Multimerin-2 OS=Mus musc	MMRN2_MOUSE	only in AngII
Four and a half LIM domains protein 3 OS	FHL3_MOUSE	only in AngII

Myristoylated alanine-rich C-kinase subst	MARCS_MOUSE	only in AngII
Voltage-dependent anion-selective chann	VDAC2_MOUSE	only in AngII
Plexin domain-containing protein 2 OS=M	PXDC2_MOUSE	only in AngII
Cluster of ADP/ATP translocase 1 OS=Mu	ADT1_MOUSE	only in AngII
ATP synthase subunit d, mitochondrial OS	ATP5H_MOUSE	only in AngII
Coatomer subunit delta OS=Mus musculu	COPD_MOUSE	only in AngII
Mannose-1-phosphate guanyltransferase	GMPPB_MOUSE	only in AngII
L-lactate dehydrogenase B chain OS=Mus	LDHB_MOUSE	only in AngII
Multiple epidermal growth factor-like do	MEG10_MOUSE	only in AngII
Peroxiredoxin-4 OS=Mus musculus OX=10	PRDX4_MOUSE	only in AngII
Cytochrome b-c1 complex subunit 1, mito	QCR1_MOUSE	only in AngII
Alpha-soluble NSF attachment protein OS	SNAA_MOUSE	only in AngII
Syntaxin-7 OS=Mus musculus OX=10090 (	STX7_MOUSE	only in AngII
Vasorin OS=Mus musculus OX=10090 GN	VASN_MOUSE	only in AngII
PDZ and LIM domain protein 7 OS=Mus m	PDLI7_MOUSE	only in AngII
3'(2'),5'-bisphosphate nucleotidase 1 OS=	BPNT1_MOUSE	only in AngII
Collagen alpha-2(VI) chain OS=Mus musc	CO6A2_MOUSE	only in AngII
Protein FAM198B OS=Mus musculus OX=	F198B_MOUSE	only in AngII
Inosine triphosphate pyrophosphatase OS	ITPA_MOUSE	only in AngII
Pyridoxal phosphate homeostasis protein	PLPHP_MOUSE	only in AngII
60S ribosomal protein L13a OS=Mus mus	RL13A_MOUSE	only in AngII
Citrate synthase, mitochondrial OS=Mus	CISY_MOUSE	only in AngII
Ubiquitin-fold modifier-conjugating enzyr	UFC1_MOUSE	only in AngII
Collectin-12 OS=Mus musculus OX=10090	COL12_MOUSE	only in AngII
Cytochrome c oxidase subunit 4 isoform 1	COX41_MOUSE	only in AngII
Heat shock 70 kDa protein 13 OS=Mus mu	HSP13_MOUSE	only in AngII
Gamma-aminobutyric acid receptor-asso	GBRAP_MOUSE	only in AngII
Isocitrate dehydrogenase [NADP], mitoch	IDHP_MOUSE	only in AngII
Inhibin beta B chain OS=Mus musculus O	INHBB_MOUSE	only in AngII
Macrophage mannose receptor 1 OS=Mu	MRC1_MOUSE	only in AngII
S-methyl-5'-thioadenosine phosphorylase	MTAP_MOUSE	only in AngII
Serine/threonine-protein phosphatase 2A	PTPA_MOUSE	only in AngII
UV excision repair protein RAD23 homolo	RD23B_MOUSE	only in AngII
Isoform 2 of Neural cell adhesion molecu	NCAM1_MOUSE	only in AngII
Isoform 2 of Amyloid-like protein 2 OS=M	APLP2_MOUSE	only in AngII
LIM and SH3 domain protein 1 OS=Mus m	LASP1_MOUSE	only in AngII
Isoform 2 of Cellular nucleic acid-binding	CNBP_MOUSE	only in AngII
Serine protease 23 OS=Mus musculus OX	PRS23_MOUSE	only in AngII
UPF0764 protein C16orf89 homolog OS=M	CP089_MOUSE	only in AngII
Semaphorin-3A OS=Mus musculus OX=10	SEM3A_MOUSE	only in AngII
Creatine kinase B-type OS=Mus musculus	KCRB_MOUSE	only in AngII
Beta-1,4-galactosyltransferase 5 OS=Mus	B4GT5_MOUSE	only in AngII
Calcium-binding protein 39 OS=Mus musc	CAB39_MOUSE	only in AngII
Transforming growth factor beta-2 propro	TGFB2_MOUSE	only in AngII
Calcium-regulated heat stable protein 1 C	CHSP1_MOUSE	only in AngII
Latexin OS=Mus musculus OX=10090 GN=	LXN_MOUSE	only in AngII
26S proteasome non-ATPase regulatory s	PSD11_MOUSE	only in AngII
40S ribosomal protein S9 OS=Mus muscu	RS9_MOUSE	only in AngII

T-complex protein 1 subunit beta OS=Mu	TCPB_MOUSE	only in AngII
Isoform 3 of Soluble calcium-activated nu	CANT1_MOUSE	only in AngII
Isoform 2 of Adenylate kinase isoenzyme	KAD1_MOUSE	only in AngII
Lysosomal alpha-glucosidase OS=Mus mu	LYAG_MOUSE	only in AngII
Phosphoserine phosphatase OS=Mus mus	SERB_MOUSE	only in AngII
Isoform 2 of Acyl-protein thioesterase 1 C	LYPA1_MOUSE	only in AngII
Isoform 2 of Elongation factor 1-delta OS	EF1D_MOUSE	only in AngII
Isoform 2 of Aly/REF export factor 2 OS=I	ALRF2_MOUSE	only in AngII
UDP-N-acetylhexosamine pyrophosphoryl	UAP1L_MOUSE	only in AngII
N(G),N(G)-dimethylarginine dimethylami	DDAH1_MOUSE	only in AngII
Myozenin-2 OS=Mus musculus OX=10090	MYOZ2_MOUSE	only in AngII
Extracellular sulfatase Sulf-2 OS=Mus mu	SULF2_MOUSE	only in AngII
Receptor-type tyrosine-protein phosphata	PTPRM_MOUSE	only in AngII
Inorganic pyrophosphatase OS=Mus musc	IPYR_MOUSE	only in AngII
N-acetylneuraminate lyase OS=Mus musc	NPL_MOUSE	only in AngII
GTP-binding protein SAR1b OS=Mus mus	SAR1B_MOUSE	only in AngII
Translin OS=Mus musculus OX=10090 GN	TSN_MOUSE	only in AngII
Myomesin 2 OS=Mus musculus OX=10090	Q14BI5_MOUSE	only in AngII
Nectin-2 OS=Mus musculus OX=10090 GN	NECT2_MOUSE	only in AngII
Oligoribonuclease, mitochondrial OS=Mu	ORN_MOUSE	only in AngII
Histidine--tRNA ligase, cytoplasmic OS=M	SYHC_MOUSE	only in AngII
60S ribosomal protein L15 OS=Mus musc	RL15_MOUSE	only in AngII
Stromelysin-3 OS=Mus musculus OX=100	MMP11_MOUSE	only in AngII
45 kDa calcium-binding protein OS=Mus r	CAB45_MOUSE	only in AngII
MLV-related proviral Env polyprotein OS=	ENV1_MOUSE	only in AngII
Matrix remodeling-associated protein 8 C	MXRA8_MOUSE	only in AngII
26S proteasome non-ATPase regulatory s	PSMD9_MOUSE	only in AngII
Ras-related protein Rab-5C OS=Mus mus	RAB5C_MOUSE	only in AngII
Arginine--tRNA ligase, cytoplasmic OS=M	SYRC_MOUSE	only in AngII
Disintegrin and metalloproteinase domai	ADA10_MOUSE	only in AngII
N(G),N(G)-dimethylarginine dimethylami	DDAH2_MOUSE	only in AngII
Pyruvate dehydrogenase E1 component su	ODPB_MOUSE	only in AngII
Ubiquitin thioesterase OTUB1 OS=Mus m	OTUB1_MOUSE	only in AngII
60S ribosomal protein L8 OS=Mus muscu	RL8_MOUSE	only in AngII
Isoform Cytoplasmic of Fumarate hydrata	FUMH_MOUSE	only in AngII
Isoform 2 of Mannan-binding lectin serine	MASP1_MOUSE	only in AngII
Isoform 2 of Protein CDV3 OS=Mus muscu	CDV3_MOUSE	only in AngII
Isoform 2 of Sacsin OS=Mus musculus OX	SACS_MOUSE	only in AngII
Isoform 3 of NSFL1 cofactor p47 OS=Mus	NSF1C_MOUSE	only in AngII
Alpha-mannosidase 2 OS=Mus musculus	MA2A1_MOUSE	only in AngII
Growth/differentiation factor 15 OS=Mus	GDF15_MOUSE	only in AngII
Isoform 2 of Roundabout homolog 4 OS=I	ROBO4_MOUSE	only in AngII
Isoform 2 of Poly(rC)-binding protein 2 OS	PCBP2_MOUSE	only in AngII
Acetyl-CoA acetyltransferase, cytosolic OS	THIC_MOUSE	only in AngII
Tolloid-like protein 1 OS=Mus musculus C	TLL1_MOUSE	only in AngII
ATP synthase subunit gamma, mitochond	ATPG_MOUSE	only in AngII
Cytochrome c oxidase subunit 2 OS=Mus	COX2_MOUSE	only in AngII
EGF-containing fibulin-like extracellular m	FBLN4_MOUSE	only in AngII

E3 ubiquitin-protein ligase NEDD4 OS=Mu	NEDD4_MOUSE	only in AngII
Serine/threonine-protein phosphatase 2A	PP2AA_MOUSE	only in AngII
40S ribosomal protein S11 OS=Mus musc	RS11_MOUSE	only in AngII
SPARC-like protein 1 OS=Mus musculus C	SPRL1_MOUSE	only in AngII
Isoform 2 of 2-oxoglutarate dehydrogena	ODO1_MOUSE	only in AngII
Adapter molecule crk OS=Mus musculus (	CRK_MOUSE	only in AngII
Isoform 2 of UTP--glucose-1-phosphate u	UGPA_MOUSE	only in AngII
Obg-like ATPase 1 OS=Mus musculus OX=	OLA1_MOUSE	only in AngII
60S ribosomal protein L21 OS=Mus musc	RL21_MOUSE	only in AngII
Disintegrin and metalloproteinase domai	ADA19_MOUSE	only in AngII
Neuropilin-1 OS=Mus musculus OX=1009(	NRP1_MOUSE	only in AngII
Caveolae-associated protein 1 OS=Mus m	CAVN1_MOUSE	only in AngII
Isoform B of Heat shock protein beta-1 O	HSPB1_MOUSE	only in AngII
Calponin-2 OS=Mus musculus OX=10090 (	CNN2_MOUSE	only in AngII
Proliferating cell nuclear antigen OS=Mus	PCNA_MOUSE	only in AngII
26S proteasome non-ATPase regulatory s	PSDE_MOUSE	only in AngII
REVERSED Isoform 2 of VPS9 domain-cor	VP9D1_MOUSE	only in AngII
Cluster of Ras-related protein Rab-2A OS	RAB2A_MOUSE	only in AngII
Cytosolic non-specific dipeptidase OS=Mu	CNDP2_MOUSE	only in AngII
Persulfide dioxygenase ETHE1, mitochond	ETHE1_MOUSE	only in AngII
Plastin-3 OS=Mus musculus OX=10090 GI	PLST_MOUSE	only in AngII
Serine/threonine-protein phosphatase PP	PP1A_MOUSE	only in AngII
Isoform 2 of Small glutamine-rich tetratr	SGTA_MOUSE	only in AngII
Coatomer subunit alpha OS=Mus musculu	COPA_MOUSE	only in AngII
Caprin-1 OS=Mus musculus OX=10090 GN	CAPR1_MOUSE	only in AngII
Trifunctional enzyme subunit beta, mitoc	ECHB_MOUSE	only in AngII
Mitogen-activated protein kinase 3 OS=M	MK03_MOUSE	only in AngII
ADP-ribosylation factor-like protein 3 OS=	ARL3_MOUSE	only in AngII
Coatomer subunit zeta-1 OS=Mus muscul	COPZ1_MOUSE	only in AngII
Endothelial cell-selective adhesion molec	ESAM_MOUSE	only in AngII
Importin subunit beta-1 OS=Mus musculu	IMB1_MOUSE	only in AngII
Lysosome-associated membrane glycopro	LAMP1_MOUSE	only in AngII
Profilin-1 OS=Mus musculus OX=10090 G	PROF1_MOUSE	only in AngII
RNA-binding protein 3 OS=Mus musculus	RBM3_MOUSE	only in AngII
Vacuolar protein sorting-associated prote	VPS35_MOUSE	only in AngII
Heterogeneous nuclear ribonucleoprotein	ROA1_MOUSE	only in AngII
60S ribosomal protein L11 (Fragment) OS	A2BH06_MOUSE	only in AngII
Ribonuclease 4 OS=Mus musculus OX=10	Q8C7E4_MOUSE	only in AngII
40S ribosomal protein S13 OS=Mus musc	RS13_MOUSE	only in AngII
Catalase OS=Mus musculus OX=10090 GN	CATA_MOUSE	only in AngII
Isoform PDE2A1 of cGMP-dependent 3',5	PDE2A_MOUSE	only in AngII
Peptidyl-prolyl cis-trans isomerase-like 1	PPIL1_MOUSE	only in AngII
Dynactin subunit 3 OS=Mus musculus OX=	DCTN3_MOUSE	only in AngII
Glycerol-3-phosphate phosphatase OS=M	PGP_MOUSE	only in AngII
Tyrosine--tRNA ligase, cytoplasmic OS=M	SYYC_MOUSE	only in AngII
Alpha-L-iduronidase OS=Mus musculus O	IDUA_MOUSE	only in AngII
Collagen triple helix repeat-containing pr	CTHR1_MOUSE	only in AngII
Calcyclin-binding protein OS=Mus muscul	CYBP_MOUSE	only in AngII

MOB kinase activator 1B OS=Mus muscul	MOB1B_MOUSE	only in AngII
N-acetyl-D-glucosamine kinase OS=Mus r	NAGK_MOUSE	only in AngII
Isoform B of AP-2 complex subunit alpha-	AP2A1_MOUSE	only in AngII
Small ubiquitin-related modifier 2 OS=Mu	D3Z794_MOUSE	only in AngII
T-complex protein 1 subunit gamma OS=	TCPG_MOUSE	only in AngII
Nucleotide exchange factor SIL1 OS=Mus	SIL1_MOUSE	only in AngII
60S ribosomal protein L24 OS=Mus musc	RL24_MOUSE	only in AngII
Isoform 2 of Armadillo repeat-containing	ARM10_MOUSE	only in AngII
Exostosin-2 OS=Mus musculus OX=10090	EXT2_MOUSE	only in AngII
Medium-chain specific acyl-CoA dehydrog	ACADM_MOUSE	only in AngII
Exostosin-1 OS=Mus musculus OX=10090	EXT1_MOUSE	only in AngII
Histone H1.2 OS=Mus musculus OX=1009	H12_MOUSE	only in AngII
Ribosyldihydronicotinamide dehydrogena	NQO2_MOUSE	only in AngII
PDZ and LIM domain protein 1 OS=Mus m	PDLI1_MOUSE	only in AngII
Rho-related GTP-binding protein RhoG OS	RHOG_MOUSE	only in AngII
Stathmin OS=Mus musculus OX=10090 G	STMN1_MOUSE	only in AngII
Isoform 2 of Intercellular adhesion molec	ICAM1_MOUSE	only in AngII
Isoform 2 of COP9 signalosome complex	CSN7A_MOUSE	only in AngII
Keratin, type II cytoskeletal 8 OS=Mus mu	K2C8_MOUSE	only in AngII
Protein transport protein Sec23A OS=Mus	SC23A_MOUSE	only in AngII
Ephrin-B2 OS=Mus musculus OX=10090 G	EFNB2_MOUSE	only in AngII
WNT1-inducible-signaling pathway protei	WISP1_MOUSE	only in AngII
Aspartyl aminopeptidase OS=Mus muscu	DNPEP_MOUSE	only in AngII
Dihydrolipoyllysine-residue acetyltransfer	ODP2_MOUSE	only in AngII
DNA-directed RNA polymerase II subunit	RPB7_MOUSE	only in AngII
ES1 protein homolog, mitochondrial OS=M	ES1_MOUSE	only in AngII
Isoform 2 of N-alpha-acetyltransferase 50	NAA50_MOUSE	only in AngII
Src substrate cortactin OS=Mus musculus	SRC8_MOUSE	only in AngII
Sialidase-1 OS=Mus musculus OX=10090	NEUR1_MOUSE	only in AngII
Matrix metalloproteinase-19 OS=Mus mu	MMP19_MOUSE	only in AngII
Isoform 3 of RNA polymerase-associated	CTR9_MOUSE	only in AngII
Isovaleryl-CoA dehydrogenase, mitochond	IVD_MOUSE	only in AngII
Protein MEMO1 OS=Mus musculus OX=10	MEMO1_MOUSE	only in AngII
Mitogen-activated protein kinase 1 OS=M	MK01_MOUSE	only in AngII
Guanine nucleotide-binding protein G(I)/G	GBB2_MOUSE	only in AngII
Isoamyl acetate-hydrolyzing esterase 1 ho	IAH1_MOUSE	only in AngII
Xaa-Pro dipeptidase OS=Mus musculus O	PEPD_MOUSE	only in AngII
Ras-related protein Rab-14 OS=Mus mus	MYH6_MOUSE	243,00

Identified Proteins	Accession Number	Ratio BPM5/Ctr
Fermitin family homolog 3 OS=Mus mu	URP2_MOUSE	0,00
GTP-binding protein OS=Mus musculus	Q61635_MOUSE	0,00
Nucleoprotein TPR OS=Mus musculus O	TPR_MOUSE	0,00
Arachidonate 12-lipoxygenase, 12S-type	LOX12_MOUSE	0,00
Protein kinase C OS=Mus musculus OX=	Q4VA93_MOUSE	0,00
Pyrin and HIN domain-containing protei	IFIX_MOUSE	0,00
EH domain-containing protein 4 OS=Mu	EHD4_MOUSE	0,00
Isoform 2 of Phosphatidylinositol 3,4,5-t	SHIP1_MOUSE	0,00
Calpain-2 catalytic subunit OS=Mus mus	CAN2_MOUSE	0,00
SWI/SNF-related matrix-associated acti	SMCA5_MOUSE	0,00
Platelet factor 4 OS=Mus musculus OX=	PLF4_MOUSE	0,00
Ly6g6f protein OS=Mus musculus OX=10	B2RXM6_MOUSE	0,00
Nuclear mitotic apparatus protein 1 OS=	NUMA1_MOUSE	0,00
CCR4-NOT transcription complex subuni	CNOT9_MOUSE	0,00
EH domain-containing protein 1 OS=Mu	EHD1_MOUSE	0,00
V-type proton ATPase subunit B, brain is	VATB2_MOUSE	0,00
Immunoglobulin kappa variable 4-57 (Fr	A0A0A6YYE7_MOUSE	0,00
Septin-7 OS=Mus musculus OX=10090 G	SEPT7_MOUSE	0,00
Complement C4-B OS=Mus musculus O	CO4B_MOUSE	0,00
Very-long-chain (3R)-3-hydroxyacyl-CoA	HACD3_MOUSE	0,00
Bifunctional glutamate/proline--tRNA li	SYEP_MOUSE	0,00
Tubulin beta-1 chain OS=Mus musculus	TBB1_MOUSE	0,00
Copine-3 OS=Mus musculus OX=10090 G	CPNE3_MOUSE	0,00
REVERSED Titin OS=Mus musculus OX=	TITIN_MOUSE	0,00
Isoform HK1 of Hexokinase-1 OS=Mus m	HXK1_MOUSE	0,00
Coronin-1A OS=Mus musculus OX=1009	COR1A_MOUSE	0,00
Peptidyl-prolyl cis-trans isomerase F, m	PPIF_MOUSE	0,00
Isoform 2 of Tyrosine-protein phosphata	PTN6_MOUSE	0,00
Isoform 2 of Heterogeneous nuclear ribo	HNRPK_MOUSE	0,00
Malectin OS=Mus musculus OX=10090 G	MLEC_MOUSE	0,00
NADH dehydrogenase [ubiquinone] 1 alp	NDUA8_MOUSE	0,00
Lamin-B2 OS=Mus musculus OX=10090	LMNB2_MOUSE	0,00
Trafficking protein particle complex sub	TPPC3_MOUSE	0,00
Isoform 2 of Programmed cell death pro	PDCD6_MOUSE	0,00
Coagulation factor XIII A chain OS=Mus	F13A_MOUSE	0,00
Isoform Delta of Lamina-associated poly	LAP2B_MOUSE	0,00
Pre-mRNA-processing-splicing factor 8 G	PRP8_MOUSE	0,00
Polypyrimidine tract-binding protein 1 O	PTBP1_MOUSE	0,00
Isoform 2 of Calcium/calmodulin-depen	KCC2D_MOUSE	0,00
Protein ERGIC-53 OS=Mus musculus OX	LMAN1_MOUSE	0,00
Isoform 2 of Heterogeneous nuclear ribo	HNRPU_MOUSE	0,00
Protein NipSnap homolog 3B OS=Mus m	NPS3B_MOUSE	0,00
Endoplasmic reticulum resident protein	ERP44_MOUSE	0,00
Metalloproteinase inhibitor 3 OS=Mus r	TIMP3_MOUSE	0,00
Coatomer subunit beta' OS=Mus muscu	COPB2_MOUSE	0,00
Isoform CNPI of 2',3'-cyclic-nucleotide 3	CN37_MOUSE	0,00
NADH dehydrogenase [ubiquinone] flavo	NDUV2_MOUSE	0,00

Stress-70 protein, mitochondrial OS=Mu	GRP75_MOUSE	0,00
Trafficking protein particle complex sub	TPPC4_MOUSE	0,00
Isoform 2 of Inter alpha-trypsin inhibito	ITIH4_MOUSE	0,00
Nucleophosmin OS=Mus musculus OX=1	NPM_MOUSE	0,00
Acyl-coenzyme A thioesterase 2, mitoch	ACOT2_MOUSE	0,00
Dolichyl-diphosphooligosaccharide--prot	OST48_MOUSE	0,00
Isoform SERCA3A of Sarcoplasmic/endo	AT2A3_MOUSE	0,00
26S proteasome non-ATPase regulatory	PSMD2_MOUSE	0,00
Fibroblast growth factor 2 OS=Mus mus	FGF2_MOUSE	0,00
BTB/POZ domain-containing protein KC	KCD12_MOUSE	0,00
Complement C3 OS=Mus musculus OX=	CO3_MOUSE	0,00
Isoform VEGF-1 of Vascular endothelial	VEGFA_MOUSE	0,00
Calpain small subunit 1 OS=Mus muscu	CPNS1_MOUSE	0,00
Isoform 2 of Tenascin-N OS=Mus muscu	TENN_MOUSE	0,00
Isoform 2 of Serine/threonine-protein p	PGAM5_MOUSE	0,00
Ras GTPase-activating-like protein IQGA	IQGA1_MOUSE	0,00
Dihydrolipoyllysine-residue succinyltrans	ODO2_MOUSE	0,00
Cytoskeleton-associated protein 4 OS=M	CKAP4_MOUSE	0,00
Isoform Short of H-2 class II histocompa	HG2A_MOUSE	0,00
Tartrate-resistant acid phosphatase typ	PPA5_MOUSE	0,00
Isoform Smooth muscle of Myosin light	MYL6_MOUSE	0,00
Integral membrane protein 2B OS=Mus	ITM2B_MOUSE	0,00
Isoform 2 of Tyrosine-protein phosphata	PTN11_MOUSE	0,00
Immunoglobulin superfamily member 8	IGSF8_MOUSE	0,00
Clathrin heavy chain 1 OS=Mus musculu	CLH1_MOUSE	0,00
Platelet glycoprotein IX OS=Mus muscul	GPIX_MOUSE	0,00
ELAV-like protein 1 OS=Mus musculus C	ELAV1_MOUSE	0,00
Apolipoprotein A-I OS=Mus musculus O	APOA1_MOUSE	0,00
Cluster of Ras-related protein Rap-1b O	RAP1B_MOUSE	0,00
Arginine-rich, mutated in early stage tu	Q3TMX5_MOUSE	0,00
Cluster of Ras-related protein Rab-1B O	RAB1B_MOUSE	0,00
Dihydrolipoyl dehydrogenase, mitochon	DLDH_MOUSE	0,00
Thioredoxin domain-containing protein	TXD12_MOUSE	0,00
Hydroxyacyl-coenzyme A dehydrogenase	HCDH_MOUSE	0,00
Cluster of ADP-ribosylation factor 1 OS=	ARF1_MOUSE	0,00
Spectrin beta chain, non-erythrocytic 1 (	SPTB2_MOUSE	0,00
Isoform Short of Latent-transforming gr	LTBP1_MOUSE	0,00
Mesothelin OS=Mus musculus OX=1009	MSLN_MOUSE	0,00
Complement C1q tumor necrosis factor-	C1QT5_MOUSE	0,00
Titin OS=Mus musculus OX=10090 GN=	TITIN_MOUSE	0,00
Latent-transforming growth factor beta	LTBP2_MOUSE	0,00
40S ribosomal protein S18 OS=Mus mus	RS18_MOUSE	0,00
Glutathione peroxidase 3 OS=Mus musc	GPX3_MOUSE	0,00
Delta(3,5)-Delta(2,4)-dienoyl-CoA isome	ECH1_MOUSE	0,00
Proteasome subunit beta type-3 OS=Mu	PSB3_MOUSE	0,00
Destrin OS=Mus musculus OX=10090 GI	DEST_MOUSE	0,00
Ras-related protein Rab-11B OS=Mus m	RB11B_MOUSE	0,00
Cluster of Tropomyosin alpha-1 chain O	TPM1_MOUSE	0,00

Ras suppressor protein 1 OS=Mus musc	RSU1_MOUSE	0,00
Protein-lysine 6-oxidase OS=Mus muscu	LYOX_MOUSE	0,00
Translationally-controlled tumor protein	TCTP_MOUSE	0,00
Protein CTLA-2-alpha OS=Mus musculus	CTL2A_MOUSE	0,00
Angiopoietin-related protein 2 OS=Mus	ANGL2_MOUSE	0,00
Angiopoietin-1 OS=Mus musculus OX=1	ANGP1_MOUSE	0,00
Tropomodulin-3 OS=Mus musculus OX=	TMOD3_MOUSE	0,01
ATP synthase subunit alpha, mitochondr	ATPA_MOUSE	0,01
Vesicular integral-membrane protein VI	LMAN2_MOUSE	0,04
Myosin-9 OS=Mus musculus OX=10090	MYH9_MOUSE	0,04
von Willebrand factor OS=Mus musculu	VWF_MOUSE	0,04
Adenylyl cyclase-associated protein 1 OS	CAP1_MOUSE	0,04
60 kDa heat shock protein, mitochondria	CH60_MOUSE	0,05
Actin-related protein 2/3 complex subur	ARPC3_MOUSE	0,05
Talin-1 OS=Mus musculus OX=10090 GN	TLN1_MOUSE	0,05
Multimerin-1 OS=Mus musculus OX=10(	MMRN1_MOUSE	0,05
Superoxide dismutase [Mn], mitochondr	SODM_MOUSE	0,05
Actin-related protein 2/3 complex subur	ARPC2_MOUSE	0,05
Fibrinogen alpha chain OS=Mus musculu	FIBA_MOUSE	0,06
Isoform 2 of MICOS complex subunit Mi	MIC60_MOUSE	0,06
Coagulation factor V OS=Mus musculus	FA5_MOUSE	0,08
Enoyl-CoA delta isomerase 1, mitochond	ECI1_MOUSE	0,08
Endonuclease domain-containing 1 prot	ENDD1_MOUSE	0,08
Cell division control protein 42 homolog	CDC42_MOUSE	0,08
Isoform 2 of Band 3 anion transport pro	B3AT_MOUSE	0,08
Fibrinogen beta chain OS=Mus musculu	FIBB_MOUSE	0,09
Eukaryotic translation initiation factor 5	IF5A1_MOUSE	0,10
Thrombospondin-2 OS=Mus musculus O	TSP2_MOUSE	0,11
Gamma-interferon-inducible lysosomal	GILT_MOUSE	0,11
Actin-related protein 2/3 complex subur	ARPC5_MOUSE	0,12
Fibrinogen gamma chain OS=Mus musc	FIBG_MOUSE	0,12
ATP synthase subunit beta, mitochondri	ATPB_MOUSE	0,12
Actin-related protein 2/3 complex subur	ARPC4_MOUSE	0,13
Lysosomal acid lipase/cholesteryl ester	LICH_MOUSE	0,13
Inositol monophosphatase 1 OS=Mus m	IMPA1_MOUSE	0,13
Glucosamine-6-phosphate isomerase 1	GNPI1_MOUSE	0,13
26S proteasome non-ATPase regulatory	PSMD8_MOUSE	0,13
Aspartate aminotransferase, mitochond	AATM_MOUSE	0,13
Gelsolin OS=Mus musculus OX=10090 G	GELS_MOUSE	0,13
Protein SEC13 homolog OS=Mus muscu	SEC13_MOUSE	0,13
Lamin-B1 OS=Mus musculus OX=10090	LMNB1_MOUSE	0,14
Filamin-A OS=Mus musculus OX=10090	FLNA_MOUSE	0,14
Glia-derived nexin OS=Mus musculus O	GDN_MOUSE	0,14
Macrophage metalloelastase OS=Mus r	MMP12_MOUSE	0,14
Protein-glutamine gamma-glutamyltran	TGM2_MOUSE	0,14
Peptidyl-prolyl cis-trans isomerase B OS	PPIB_MOUSE	0,14
Hypoxia up-regulated protein 1 OS=Mus	HYOU1_MOUSE	0,14
Protein FAM3C OS=Mus musculus OX=1	FAM3C_MOUSE	0,14

Cathepsin S OS=Mus musculus OX=1009	CATS_MOUSE	0,15
GrpE protein homolog 1, mitochondrial	GRPE1_MOUSE	0,15
EMILIN-1 OS=Mus musculus OX=10090	EMIL1_MOUSE	0,16
Urokinase-type plasminogen activator C	UROK_MOUSE	0,16
Cluster of Isoform 2 of Tropomyosin alp	TPM3_MOUSE	0,17
Protein CREG1 OS=Mus musculus OX=1(	CREG1_MOUSE	0,17
Ubiquitin-60S ribosomal protein L40 OS	RL40_MOUSE	0,17
Chitinase-like protein 3 OS=Mus muscul	CHIL3_MOUSE	0,17
Marginal zone B- and B1-cell-specific pr	MZB1_MOUSE	0,18
Thioredoxin-dependent peroxide reducta	PRDX3_MOUSE	0,18
Collagen alpha-1(III) chain OS=Mus mus	CO3A1_MOUSE	0,18
Isoform Short of Collagen alpha-1(XI) ch	COBA1_MOUSE	0,18
Platelet glycoprotein Ib beta chain OS=M	GP1BB_MOUSE	0,19
F-actin-capping protein subunit alpha-2	CAZA2_MOUSE	0,19
Calreticulin OS=Mus musculus OX=1009	CALR_MOUSE	0,20
IST1 homolog OS=Mus musculus OX=10	IST1_MOUSE	0,20
Proteasome subunit alpha type-4 OS=M	PSA4_MOUSE	0,20
Transforming growth factor beta-1 prop	TGFB1_MOUSE	0,22
Isoform 2 of Spectrin alpha chain, non-e	SPTN1_MOUSE	0,22
Desmin OS=Mus musculus OX=10090 G	DESM_MOUSE	0,22
Purine nucleoside phosphorylase OS=Mu	PNPH_MOUSE	0,23
NPC intracellular cholesterol transporte	NPC2_MOUSE	0,23
Rho GDP-dissociation inhibitor 2 OS=Mu	GDIR2_MOUSE	0,23
Cleavage and polyadenylation specificity	CPSF5_MOUSE	0,24
Isoform 2 of F-actin-capping protein sub	CAPZB_MOUSE	0,24
L-lactate dehydrogenase A chain OS=Mu	LDHA_MOUSE	0,24
Myosin regulatory light chain 12B OS=M	ML12B_MOUSE	0,25
Vasodilator-stimulated phosphoprotein	VASP_MOUSE	0,25
Isoform IGF-IB of Insulin-like growth fac	IGF1_MOUSE	0,25
Cluster of Isoform 2 of Heterogeneous r	HNRPF_MOUSE	0,25
Phosphatidylinositol transfer protein alp	PIPNA_MOUSE	0,25
Isoform PLEC-1I of Plectin OS=Mus mus	PLEC_MOUSE	0,25
Malate dehydrogenase, mitochondrial C	MDHM_MOUSE	0,26
Collagen, type VI, alpha 3 OS=Mus musc	E9PWQ3_MOUSE	0,26
F-actin-capping protein subunit alpha-1	CAZA1_MOUSE	0,27
Peptidyl-prolyl cis-trans isomerase C OS	PPIC_MOUSE	0,28
Deoxyribonuclease-2-alpha OS=Mus mu	DNS2A_MOUSE	0,29
Heme oxygenase 1 OS=Mus musculus C	HMOX1_MOUSE	0,29
Complement C1q subcomponent subuni	C1QC_MOUSE	0,29
Protein disulfide-isomerase OS=Mus mu	PDIA1_MOUSE	0,30
Complement C1q subcomponent subuni	C1QA_MOUSE	0,30
Metalloproteinase inhibitor 2 OS=Mus r	Q6PI17_MOUSE	0,30
Cluster of Elongation factor 1-alpha 1 O	EF1A1_MOUSE	0,31
Cluster of Actin, cytoplasmic 1 OS=Mus	ACTB_MOUSE	0,33
Complement C1q subcomponent subuni	C1QB_MOUSE	0,33
Component of Sp100-rs OS=Mus muscu	CSPRS_MOUSE	0,33
Actin-related protein 3 OS=Mus muscul	ARP3_MOUSE	0,33
Proteasome subunit alpha type-2 OS=M	PSA2_MOUSE	0,33

Germinal-center associated nuclear pro	GANP_MOUSE	0,33
Putative phospholipase B-like 2 OS=Mus	PLBL2_MOUSE	0,33
Isoform 2 of Neutral alpha-glucosidase	GANAB_MOUSE	0,33
Cluster of Histone H2A type 1-B OS=Mus	H2A1B_MOUSE	0,35
Cystatin-C OS=Mus musculus OX=10090	CYTC_MOUSE	0,35
Isoform 2 of Trem-like transcript 1 prote	TRML1_MOUSE	0,36
Ferritin heavy chain OS=Mus musculus (	FRIH_MOUSE	0,36
Integrin alpha-IIb OS=Mus musculus OX	ITA2B_MOUSE	0,36
Cathepsin L1 OS=Mus musculus OX=100	CATL1_MOUSE	0,36
Pigment epithelium-derived factor OS=I	PEDF_MOUSE	0,36
Ferritin OS=Mus musculus OX=10090 G	Q9CPX4_MOUSE	0,36
Antithrombin-III OS=Mus musculus OX=	ANT3_MOUSE	0,37
40S ribosomal protein S4, X isoform OS	RS4X_MOUSE	0,38
C-C motif chemokine 2 OS=Mus muscul	CCL2_MOUSE	0,38
Delta-aminolevulinic acid dehydratase (	HEM2_MOUSE	0,40
Flavin reductase (NADPH) OS=Mus mus	BLVRB_MOUSE	0,40
Isoform 2 of Immunoglobulin heavy con	IGHM_MOUSE	0,40
Fibrillin-1 OS=Mus musculus OX=10090	FBN1_MOUSE	0,40
Nucleoside diphosphate kinase B OS=M	NDKB_MOUSE	0,40
Cluster of Glyceraldehyde-3-phosphate	G3P_MOUSE	0,41
Ganglioside GM2 activator OS=Mus mu	SAP3_MOUSE	0,41
Rab GDP dissociation inhibitor beta OS=	GDIB_MOUSE	0,41
Apolipoprotein E OS=Mus musculus OX=	APOE_MOUSE	0,41
Transaldolase OS=Mus musculus OX=10	TALDO_MOUSE	0,41
Endoplasmic reticulum resident protein	ERP29_MOUSE	0,42
Proteasome subunit alpha type-3 OS=M	PSA3_MOUSE	0,42
H-2 class I histocompatibility antigen, K-	G3UXW2_MOUSE	0,43
Rho GDP-dissociation inhibitor 1 OS=Mu	GDIR1_MOUSE	0,43
Serum albumin OS=Mus musculus OX=1	ALBU_MOUSE	0,43
Tropomyosin alpha-4 chain OS=Mus mu	TPM4_MOUSE	0,44
Isoform 2 of Sorcin OS=Mus musculus C	SORCN_MOUSE	0,44
Proteasome subunit beta type-6 OS=Mu	PSB6_MOUSE	0,44
Collagen alpha-1(V) chain OS=Mus mus	CO5A1_MOUSE	0,44
cont|001|sp|ALBU_BOVIN|		0,45
Carbonic anhydrase 2 OS=Mus musculus	CAH2_MOUSE	0,46
Renin receptor OS=Mus musculus OX=1	RENR_MOUSE	0,46
N(4)-(beta-N-acetylglucosaminyl)-L-asp	ASPG_MOUSE	0,46
Peroxiredoxin-2 OS=Mus musculus OX=1	PRDX2_MOUSE	0,46
Cluster of Pyruvate kinase PKM OS=Mus	KPYM_MOUSE	0,46
Isoform 2 of Galectin-9 OS=Mus muscu	LEG9_MOUSE	0,50
Dipeptidyl peptidase 1 OS=Mus musculu	CATC_MOUSE	0,50
Cluster of Phosphoglycerate mutase 1 C	PGAM1_MOUSE	0,50
S-formylglutathione hydrolase OS=Mus	H3BKH6_MOUSE	0,50
Beta-glucuronidase OS=Mus musculus (	BGLR_MOUSE	0,50
Isoform 2 of Stabilin-1 OS=Mus musculu	STAB1_MOUSE	0,50
Isoform 2 of Vascular endothelial growt	VEGFC_MOUSE	0,50
Growth factor receptor-bound protein 2	GRB2_MOUSE	0,50
Proteasome subunit beta type-4 OS=Mu	PSB4_MOUSE	0,50

Cathepsin O OS=Mus musculus OX=100	CATO_MOUSE	0,50
Cysteine and glycine-rich protein 1 OS=M	CSRP1_MOUSE	0,50
Ras-related C3 botulinum toxin substrat	RAC1_MOUSE	0,50

Identified Proteins	Accession Number	Ratio BPM5/Ctr
Isoform 2 of Fibulin-2 OS=Mus musculus OX=	FBLN2_MOUSE	1,97
Pyridoxal kinase OS=Mus musculus OX=10090	PDXK_MOUSE	2,00
6-phosphogluconate dehydrogenase, decarbox	6PGD_MOUSE	2,00
Plasminogen activator inhibitor 1 OS=Mus mu	G5E899_MOUSE	2,03
Connective tissue growth factor OS=Mus mus	CTGF_MOUSE	2,06
cont|004|sp|K1C9_HUMAN|	cont|004|sp	2,06
Insulin-like growth factor-binding protein 4 OS	IBP4_MOUSE	2,11
Follistatin-related protein 1 OS=Mus musculu	FSTL1_MOUSE	2,13
Cluster of Meteorin-like protein OS=Mus mus	METRL_MOUSE	2,22
Isoform II of Gamma-glutamyl hydrolase OS=	GGH_MOUSE	2,25
Beta-2-microglobulin OS=Mus musculus OX=1	B2MG_MOUSE	2,27
Secreted frizzled-related protein 2 OS=Mus m	SFRP2_MOUSE	2,33
Galectin-3-binding protein OS=Mus musculus	LG3BP_MOUSE	2,33
Procollagen-lysine,2-oxoglutarate 5-dioxygena	PLOD1_MOUSE	2,43
Carbonyl reductase [NADPH] 1 OS=Mus muscu	CBR1_MOUSE	2,50
Tripeptidyl-peptidase 1 OS=Mus musculus OX	TPP1_MOUSE	2,56
Retinoid-inducible serine carboxypeptidase OS	RISC_MOUSE	2,67
Phosphoglycerate kinase 1 OS=Mus musculus	PGK1_MOUSE	2,73
72 kDa type IV collagenase OS=Mus musculus	MMP2_MOUSE	2,73
Amyloid-beta A4 protein OS=Mus musculus O	A4_MOUSE	2,80
Protein CYR61 OS=Mus musculus OX=10090 G	CYR61_MOUSE	2,80
Annexin A2 OS=Mus musculus OX=10090 GN=	ANXA2_MOUSE	2,88
Interleukin-18-binding protein OS=Mus muscu	I18BP_MOUSE	3,00
Cluster of Heat shock protein HSP 90-beta OS=	HS90B_MOUSE	3,00
Cluster of Alpha-actinin-4 OS=Mus musculus (	ACTN4_MOUSE	3,00
Testin-2 OS=Mus musculus OX=10090 PE=2 S	TEST2_MOUSE	3,00
Poly(rC)-binding protein 1 OS=Mus musculus (	PCBP1_MOUSE	3,00
Bone morphogenetic protein 1 OS=Mus musc	BMP1_MOUSE	3,00
Cluster of Glutathione S-transferase Mu 1 OS	GSTM1_MOUSE	3,00
Dihydropyrimidinase-related protein 2 OS=Mu	DPYL2_MOUSE	3,00
Collagen alpha-2(IV) chain OS=Mus musculus	CO4A2_MOUSE	3,17
Glutathione S-transferase omega-1 OS=Mus	GSTO1_MOUSE	3,25
Synaptic vesicle membrane protein VAT-1 hor	VAT1_MOUSE	3,38
Cadherin-11 OS=Mus musculus OX=10090 GN	CAD11_MOUSE	4,00
Peroxidasin homolog OS=Mus musculus OX=1	PXDN_MOUSE	4,00
Clusterin OS=Mus musculus OX=10090 GN=Cl	CLUS_MOUSE	4,61
Phospholipase D3 OS=Mus musculus OX=1009	PLD3_MOUSE	4,67
Endothelial protein C receptor OS=Mus muscu	EPCR_MOUSE	5,00
Isoform LAMP-2B of Lysosome-associated me	LAMP2_MOUSE	5,00
Aspartate aminotransferase, cytoplasmic OS=	AATC_MOUSE	5,00
Thioredoxin-like protein 1 OS=Mus musculus (	TXNL1_MOUSE	5,00
Isocitrate dehydrogenase [NADP] cytoplasmic	IDHC_MOUSE	5,40
Collagen alpha-1(IV) chain OS=Mus musculus	CO4A1_MOUSE	5,50
Cluster of Heat shock 70 kDa protein 4 OS=Mu	HSP74_MOUSE	6,00
Phospholipid transfer protein OS=Mus muscul	PLTP_MOUSE	6,00
Fascin OS=Mus musculus OX=10090 GN=Fscn	FSCN1_MOUSE	7,00
Carboxypeptidase Q OS=Mus musculus OX=10	CBPQ_MOUSE	8,00

Insulin-like growth factor-binding protein 3 OS	IBP3_MOUSE	8,67
Inactive tyrosine-protein kinase 7 OS=Mus mu	PTK7_MOUSE	9,00
Ribonuclease inhibitor OS=Mus musculus OX=	RINI_MOUSE	9,00
Adipocyte enhancer-binding protein 1 OS=Mu	AEBP1_MOUSE	9,00
Collagen alpha-2(V) chain OS=Mus musculus	CO5A2_MOUSE	12,50
Isoform 2 of Sulfhydryl oxidase 1 OS=Mus mu	QSOX1_MOUSE	12,67
Cadherin-5 OS=Mus musculus OX=10090 GN=	CADH5_MOUSE	69,00
Filamin-B OS=Mus musculus OX=10090 GN=F	FLNB_MOUSE	Only in Bmp5
Endothelial cell-specific molecule 1 OS=Mus r	ESM1_MOUSE	Only in Bmp5
Myosin light chain 3 OS=Mus musculus OX=10	MYL3_MOUSE	Only in Bmp5
Vascular cell adhesion protein 1 OS=Mus mus	VCAM1_MOUSE	Only in Bmp5
Isoform 2 of Cell surface glycoprotein MUC18	MUC18_MOUSE	Only in Bmp5
Beta-galactosidase OS=Mus musculus OX=10	BGAL_MOUSE	Only in Bmp5
Isoform 2 of Lactadherin OS=Mus musculus O	MFGM_MOUSE	Only in Bmp5
Complement component C1q receptor OS=Mu	C1QR1_MOUSE	Only in Bmp5
Transitional endoplasmic reticulum ATPase O	TERA_MOUSE	Only in Bmp5
60S acidic ribosomal protein P0 OS=Mus mus	RLA0_MOUSE	Only in Bmp5
Coiled-coil domain-containing protein 80 OS=	CCD80_MOUSE	Only in Bmp5
Tyrosine-protein kinase receptor Tie-1 OS=Mu	TIE1_MOUSE	Only in Bmp5
Fibulin-5 OS=Mus musculus OX=10090 GN=Fb	FBLN5_MOUSE	Only in Bmp5
Isoform 2 of Calsyntenin-1 OS=Mus musculus	CSTN1_MOUSE	Only in Bmp5
Phosphoserine aminotransferase OS=Mus mu	SERC_MOUSE	Only in Bmp5
Plasmalemma vesicle-associated protein OS=	PLVAP_MOUSE	Only in Bmp5
Complement C1s-A subcomponent OS=Mus m	CS1A_MOUSE	Only in Bmp5
Calcium-activated chloride channel regulator	CA3A1_MOUSE	Only in Bmp5
Isoform 2 of Platelet endothelial cell adhesior	PECA1_MOUSE	Only in Bmp5
Vitamin K-dependent protein S OS=Mus musc	PROS_MOUSE	Only in Bmp5
40S ribosomal protein S5 (Fragment) OS=Mus	D3YYM6_MOUSE	Only in Bmp5
Cluster of H-2 class I histocompatibility antige	HA11_MOUSE	Only in Bmp5
Aldose reductase-related protein 2 OS=Mus m	ALD2_MOUSE	Only in Bmp5
Isoform 3 of Programmed cell death 6-interac	PDC6I_MOUSE	Only in Bmp5
NK13 OS=Mus musculus OX=10090 GN=Serpi	O08804_MOUSE	Only in Bmp5
Macrophage colony-stimulating factor 1 OS=M	CSF1_MOUSE	Only in Bmp5
Lysyl oxidase homolog 3 OS=Mus musculus O	LOXL3_MOUSE	Only in Bmp5
Spermidine synthase OS=Mus musculus OX=1	SPEE_MOUSE	Only in Bmp5
Low-density lipoprotein receptor OS=Mus mus	LDLR_MOUSE	Only in Bmp5
Selenoprotein P OS=Mus musculus OX=10090	SEPP1_MOUSE	Only in Bmp5
Isoform 2 of Heterogeneous nuclear ribonucle	ROA2_MOUSE	Only in Bmp5
Calumenin OS=Mus musculus OX=10090 GN=	CALU_MOUSE	Only in Bmp5
Heterogeneous nuclear ribonucleoprotein A/B	ROAA_MOUSE	Only in Bmp5
Plasma protease C1 inhibitor OS=Mus muscul	IC1_MOUSE	Only in Bmp5
Lysyl oxidase homolog 1 OS=Mus musculus O	LOXL1_MOUSE	Only in Bmp5
Angiotensin-converting enzyme OS=Mus mus	ACE_MOUSE	Only in Bmp5
Isoform Beta of Tissue factor pathway inhibit	TFPI1_MOUSE	Only in Bmp5
Ceroid-lipofuscinosis neuronal protein 5 homo	CLN5_MOUSE	Only in Bmp5
Macrophage colony-stimulating factor 1 recep	CSF1R_MOUSE	Only in Bmp5
Zyxin OS=Mus musculus OX=10090 GN=Zyx Pl	ZYX_MOUSE	Only in Bmp5
Isoform A0 of Neuropilin-2 OS=Mus musculus	NRP2_MOUSE	Only in Bmp5

Angiopoietin-2 OS=Mus musculus OX=10090 (	ANGP2_MOUSE	Only in Bmp5

Identified Proteins	Accession Number	Ratio AngII/Bmp5
Glia-derived nexin OS=Mus musculus (	GDN_MOUSE	2,00
Endoplasmin OS=Mus musculus OX=1(	ENPL_MOUSE	2,00
Complement C1s-A subcomponent OS	CS1A_MOUSE	2,00
Ribonuclease inhibitor OS=Mus muscu	RINI_MOUSE	2,00
Isoform 3 of Programmed cell death 6	PDC6I_MOUSE	2,00
Polypyrimidine tract-binding protein 1	PTBP1_MOUSE	2,00
Chloride intracellular channel protein 4	CLIC4_MOUSE	2,00
Angiotensin-converting enzyme OS=M	ACE_MOUSE	2,00
Heme-binding protein 1 OS=Mus musc	HEBP1_MOUSE	2,00
Cadherin-11 OS=Mus musculus OX=10	CAD11_MOUSE	2,00
Delta-aminolevulinic acid dehydratase	HEM2_MOUSE	2,00
Isoform 2 of 4F2 cell-surface antigen I	4F2_MOUSE	2,00
Four and a half LIM domains protein 3	FHL3_MOUSE	2,00
Isoform Cytoplasmic of Glutathione re	GSHR_MOUSE	2,00
Isoform 2 of Aly/REF export factor 2 O	ALRF2_MOUSE	2,00
Receptor-type tyrosine-protein phosph	PTPRM_MOUSE	2,00
Monocyte differentiation antigen CD14	CD14_MOUSE	2,00
N-acetylneuraminate lyase OS=Mus m	NPL_MOUSE	2,00
Histidine--tRNA ligase, cytoplasmic OS	SYHC_MOUSE	2,00
Cluster of Caldesmon 1 OS=Mus musc	E9QA16_MOUSE	2,00
Ubiquitin thioesterase OTUB1 OS=Mu	OTUB1_MOUSE	2,00
60S ribosomal protein L8 OS=Mus mus	RL8_MOUSE	2,00
Isoform 2 of Sacsin OS=Mus musculus	SACS_MOUSE	2,00
Isoform 3 of NSFL1 cofactor p47 OS=M	NSF1C_MOUSE	2,00
Alpha-mannosidase 2 OS=Mus muscu	MA2A1_MOUSE	2,00
Growth/differentiation factor 15 OS=M	GDF15_MOUSE	2,00
Acetyl-CoA acetyltransferase, cytosolic	THIC_MOUSE	2,00
EGF-containing fibulin-like extracellula	FBLN4_MOUSE	2,00
40S ribosomal protein S11 OS=Mus m	RS11_MOUSE	2,00
Disintegrin and metalloproteinase dor	ADA19_MOUSE	2,00
Cytosolic non-specific dipeptidase OS=	CNDP2_MOUSE	2,00
Serine/threonine-protein phosphatase	PP1A_MOUSE	2,00
Trifunctional enzyme subunit beta, mi	ECHB_MOUSE	2,00
Mitogen-activated protein kinase 3 OS	MK03_MOUSE	2,00
ADP-ribosylation factor-like protein 3 (	ARL3_MOUSE	2,00
Coatomer subunit zeta-1 OS=Mus mus	COPZ1_MOUSE	2,00
Heterogeneous nuclear ribonucleoprot	ROA1_MOUSE	2,00
60S ribosomal protein L11 (Fragment)	A2BH06_MOUSE	2,00
Ribonuclease 4 OS=Mus musculus OX=	Q8C7E4_MOUSE	2,00
40S ribosomal protein S13 OS=Mus m	RS13_MOUSE	2,00
Isoform PDE2A1 of cGMP-dependent 3	PDE2A_MOUSE	2,00
Dynactin subunit 3 OS=Mus musculus	DCTN3_MOUSE	2,00
Collagen triple helix repeat-containing	CTHR1_MOUSE	2,00
Calcyclin-binding protein OS=Mus mus	CYBP_MOUSE	2,00
MOB kinase activator 1B OS=Mus mus	MOB1B_MOUSE	2,00
N-acetyl-D-glucosamine kinase OS=M	NAGK_MOUSE	2,00
Isoform B of AP-2 complex subunit alp	AP2A1_MOUSE	2,00

Nucleotide exchange factor SIL1 OS=M	SIL1_MOUSE	2,00
Medium-chain specific acyl-CoA dehyd	ACADM_MOUSE	2,00
Exostosin-1 OS=Mus musculus OX=100	EXT1_MOUSE	2,00
PDZ and LIM domain protein 1 OS=Mu	PDLI1_MOUSE	2,00
Isoform 2 of Intercellular adhesion mo	ICAM1_MOUSE	2,00
Ephrin-B2 OS=Mus musculus OX=1009	EFNB2_MOUSE	2,00
Aspartyl aminopeptidase OS=Mus mus	DNPEP_MOUSE	2,00
Synaptic vesicle membrane protein VA	VAT1_MOUSE	2,04
Isocitrate dehydrogenase [NADP] cyto	IDHC_MOUSE	2,04
Cadherin-5 OS=Mus musculus OX=100	CADH5_MOUSE	2,04
Thioredoxin domain-containing protein	TXND5_MOUSE	2,08
Chitinase-like protein 3 OS=Mus musc	CHIL3_MOUSE	2,13
Complement component C1q receptor	C1QR1_MOUSE	2,13
Cluster of Tubulin alpha-1B chain OS=	TBA1B_MOUSE	2,17
Low-density lipoprotein receptor OS=M	LDLR_MOUSE	2,17
Antithrombin-III OS=Mus musculus OX	ANT3_MOUSE	2,17
Alcohol dehydrogenase [NADP(+)] OS=	AK1A1_MOUSE	2,19
Insulin-like growth factor-binding prot	IBP3_MOUSE	2,19
Dipeptidyl peptidase 2 OS=Mus muscu	DPP2_MOUSE	2,20
Insulin-like growth factor-binding prot	IBP4_MOUSE	2,21
Pigment epithelium-derived factor OS	PEDF_MOUSE	2,23
Zyxin OS=Mus musculus OX=10090 GN	ZYX_MOUSE	2,25
Endoplasmic reticulum chaperone BiP	BIP_MOUSE	2,26
Cathepsin B OS=Mus musculus OX=10	CATB_MOUSE	2,27
Galectin-3-binding protein OS=Mus m	LG3BP_MOUSE	2,29
Integrin beta-3 OS=Mus musculus OX=	ITB3_MOUSE	2,29
Endoplasmic reticulum resident protei	ERP29_MOUSE	2,30
Multimerin-1 OS=Mus musculus OX=1	MMRN1_MOUSE	2,33
Acid sphingomyelinase-like phosphodi	ASM3A_MOUSE	2,33
Amyloid-beta A4 protein OS=Mus mus	A4_MOUSE	2,36
60S acidic ribosomal protein P0 OS=M	RLA0_MOUSE	2,38
Annexin A2 OS=Mus musculus OX=100	ANXA2_MOUSE	2,39
Serotransferrin OS=Mus musculus OX=	TRFE_MOUSE	2,40
Collagen alpha-2(V) chain OS=Mus mu	CO5A2_MOUSE	2,40
Glutaminyl-peptide cyclotransferase C	QPCT_MOUSE	2,40
Actin-related protein 2 OS=Mus muscu	ARP2_MOUSE	2,40
A disintegrin and metalloproteinase w	ATS1_MOUSE	2,40
Isoform LAMP-2B of Lysosome-associa	LAMP2_MOUSE	2,40
Phospholipid transfer protein OS=Mus	PLTP_MOUSE	2,42
Isoform 2 of Fibulin-2 OS=Mus muscu	FBLN2_MOUSE	2,43
Tripeptidyl-peptidase 1 OS=Mus musc	TPP1_MOUSE	2,43
Lysosomal protective protein OS=Mus	PPGB_MOUSE	2,44
Biglycan OS=Mus musculus OX=10090	PGS1_MOUSE	2,45
Cadherin-2 OS=Mus musculus OX=100	CADH2_MOUSE	2,45
Tubulointerstitial nephritis antigen-lik	TINAL_MOUSE	2,50
Glutathione S-transferase A4 OS=Mus	GSTA4_MOUSE	2,50
Calcium-binding protein 39 OS=Mus m	CAB39_MOUSE	2,50
Transforming growth factor beta-2 pro	TGFB2_MOUSE	2,50

Complement C1q subcomponent subu	C1QC_MOUSE	2,52
Isoform C of Prelamin-A/C OS=Mus m	LMNA_MOUSE	2,54
Cluster of Heat shock cognate 71 kDa	HSP7C_MOUSE	2,55
Chloride intracellular channel protein	CLIC1_MOUSE	2,57
Transcobalamin-2 OS=Mus musculus	TCO2_MOUSE	2,58
Fibrinogen gamma chain OS=Mus mu	FIBG_MOUSE	2,58
Apolipoprotein E OS=Mus musculus O	APOE_MOUSE	2,59
Leukocyte elastase inhibitor A OS=Mu	ILEUA_MOUSE	2,62
72 kDa type IV collagenase OS=Mus m	MMP2_MOUSE	2,63
Prolactin-2C2 OS=Mus musculus OX=1	PR2C2_MOUSE	2,65
Serine (Or cysteine) peptidase inhibitc	G3X8T9_MOUSE	2,67
Lymphocyte antigen 86 OS=Mus musc	LY86_MOUSE	2,67
Myosin-9 OS=Mus musculus OX=1009	MYH9_MOUSE	2,68
Gelsolin OS=Mus musculus OX=10090	GELS_MOUSE	2,68
cont|052|sp|P99999|IRT_SYNTH Biog	cont|052|sp	2,73
Lamin-B1 OS=Mus musculus OX=1009	LMNB1_MOUSE	2,75
Transitional endoplasmic reticulum AT	TERA_MOUSE	2,75
Lactoylglutathione lyase OS=Mus mus	LGUL_MOUSE	2,75
Retinoid-inducible serine carboxypepti	RISC_MOUSE	2,75
Isoform Beta of Tissue factor pathway	TFPI1_MOUSE	2,75
Carbonyl reductase [NADPH] 3 OS=Mu	CBR3_MOUSE	2,75
REVERSED Isoform 2 of Neuronal tyro	NYAP1_MOUSE	2,75
Alpha-N-acetylglucosaminidase OS=M	O88325_MOUSE	2,80
Actin-related protein 3 OS=Mus muscu	ARP3_MOUSE	2,80
Legumain OS=Mus musculus OX=1009	LGMN_MOUSE	2,83
Peptidyl-prolyl cis-trans isomerase A	PPIA_MOUSE	2,83
Cystatin-C OS=Mus musculus OX=1009	CYTC_MOUSE	2,83
Dipeptidyl peptidase 1 OS=Mus muscu	CATC_MOUSE	2,83
Cofilin-1 OS=Mus musculus OX=10090	COF1_MOUSE	2,85
Carboxypeptidase E OS=Mus musculus	CBPE_MOUSE	2,86
Serpin H1 OS=Mus musculus OX=1009	SERPH_MOUSE	2,86
Procollagen C-endopeptidase enhance	PCOC1_MOUSE	2,87
Cluster of Alpha-enolase OS=Mus mus	ENOA_MOUSE	2,87
Cluster of Actin, cytoplasmic 1 OS=Mu	ACTB_MOUSE	2,89
Fibrinogen beta chain OS=Mus muscu	FIBB_MOUSE	2,89
Filamin-A OS=Mus musculus OX=1009	FLNA_MOUSE	2,90
Platelet-activating factor acetylhydrola	PAFA_MOUSE	2,91
L-lactate dehydrogenase A chain OS=M	LDHA_MOUSE	2,95
Collagen alpha-1(IV) chain OS=Mus m	CO4A1_MOUSE	2,95
40S ribosomal protein SA OS=Mus mu	RSSA_MOUSE	3,00
Thioredoxin-dependent peroxide reduc	PRDX3_MOUSE	3,00
Aspartate aminotransferase, cytoplasi	AATC_MOUSE	3,00
Pre-mRNA-processing-splicing factor 8	PRP8_MOUSE	3,00
NADH dehydrogenase [ubiquinone] fla	NDUV2_MOUSE	3,00
Aldose reductase-related protein 2 OS	ALD2_MOUSE	3,00
GrpE protein homolog 1, mitochondria	GRPE1_MOUSE	3,00
Dolichyl-diphosphooligosaccharide--pr	OST48_MOUSE	3,00
26S proteasome non-ATPase regulato	PSMD2_MOUSE	3,00

Ubiquitin carboxyl-terminal hydrolase	UCHL1_MOUSE	3,00
Heat shock 70 kDa protein 13 OS=Mus	HSP13_MOUSE	3,00
Phospholipase B-like 1 OS=Mus muscu	PLBL1_MOUSE	3,00
Isoform 2 of Cellular nucleic acid-bind	CNBP_MOUSE	3,00
60S ribosomal protein L17 OS=Mus m	RL17_MOUSE	3,00
Phosphoserine phosphatase OS=Mus r	SERB_MOUSE	3,00
Lysyl oxidase homolog 2 OS=Mus mus	LOXL2_MOUSE	3,00
Isoform Alpha-6X1A of Integrin alpha-	ITA6_MOUSE	3,00
Isoform 2 of Elongation factor 1-delta	EF1D_MOUSE	3,00
Myozenin-2 OS=Mus musculus OX=10	MYOZ2_MOUSE	3,00
GTP-binding protein SAR1b OS=Mus m	SAR1B_MOUSE	3,00
Translin OS=Mus musculus OX=10090	TSN_MOUSE	3,00
Myomesin 2 OS=Mus musculus OX=10	Q14BI5_MOUSE	3,00
Nectin-2 OS=Mus musculus OX=10090	NECT2_MOUSE	3,00
Oligoribonuclease, mitochondrial OS=	ORN_MOUSE	3,00
60S ribosomal protein L15 OS=Mus m	RL15_MOUSE	3,00
26S proteasome non-ATPase regulato	PSMD9_MOUSE	3,00
Ras-related protein Rab-5C OS=Mus m	RAB5C_MOUSE	3,00
Arginine--tRNA ligase, cytoplasmic OS	SYRC_MOUSE	3,00
Disintegrin and metalloproteinase dor	ADA10_MOUSE	3,00
CCR4-NOT transcription complex subu	CNOT9_MOUSE	3,00
N(G),N(G)-dimethylarginine dimethyla	DDAH2_MOUSE	3,00
Pyruvate dehydrogenase E1 componer	ODPB_MOUSE	3,00
Isoform Cytoplasmic of Fumarate hydr	FUMH_MOUSE	3,00
Isoform 2 of Mannan-binding lectin se	MASP1_MOUSE	3,00
Isoform 2 of Protein CDV3 OS=Mus mu	CDV3_MOUSE	3,00
Tolloid-like protein 1 OS=Mus musculu	TLL1_MOUSE	3,00
Cytochrome c oxidase subunit 2 OS=M	COX2_MOUSE	3,00
E3 ubiquitin-protein ligase NEDD4 OS=	NEDD4_MOUSE	3,00
Serine/threonine-protein phosphatase	PP2AA_MOUSE	3,00
Isoform 2 of 2-oxoglutarate dehydroge	ODO1_MOUSE	3,00
Adapter molecule crk OS=Mus muscul	CRK_MOUSE	3,00
Isoform 2 of UTP--glucose-1-phosphat	UGPA_MOUSE	3,00
Isoform 2 of Hydroxyacylglutathione hy	GLO2_MOUSE	3,00
60S ribosomal protein L21 OS=Mus m	RL21_MOUSE	3,00
Neuropilin-1 OS=Mus musculus OX=10	NRP1_MOUSE	3,00
Caveolae-associated protein 1 OS=Mu	CAVN1_MOUSE	3,00
Isoform B of Heat shock protein beta-1	HSPB1_MOUSE	3,00
Calponin-2 OS=Mus musculus OX=100	CNN2_MOUSE	3,00
Proliferating cell nuclear antigen OS=M	PCNA_MOUSE	3,00
Cluster of Ras-related protein Rab-2A	RAB2A_MOUSE	3,00
Isoform 2 of Small glutamine-rich tetr	SGTA_MOUSE	3,00
Glycerol-3-phosphate phosphatase OS	PGP_MOUSE	3,00
Rho-related GTP-binding protein RhoG	RHOG_MOUSE	3,00
Isoform 2 of Sarcoplasmic/endoplasm	AT2A2_MOUSE	3,00
Angiopoietin-2 OS=Mus musculus OX=	ANGP2_MOUSE	3,02
Flavin reductase (NADPH) OS=Mus mu	BLVRB_MOUSE	3,07
Integrin alpha-IIb OS=Mus musculus O	ITA2B_MOUSE	3,11

Malate dehydrogenase, mitochondrial	MDHM_MOUSE	3,11
Cluster of Insulin-like growth factor-bi	IBP7_MOUSE	3,14
Isoform 2 of 14-3-3 protein theta OS=	1433T_MOUSE	3,15
Adipocyte enhancer-binding protein 1	AEBP1_MOUSE	3,17
Syntenin-1 OS=Mus musculus OX=100	SDCB1_MOUSE	3,17
Aldose reductase OS=Mus musculus C	ALDR_MOUSE	3,17
Complement C1q subcomponent subu	C1QB_MOUSE	3,18
6-phosphogluconate dehydrogenase, c	6PGD_MOUSE	3,20
Macrophage colony-stimulating factor	CSF1_MOUSE	3,20
Lysyl oxidase homolog 3 OS=Mus mus	LOXL3_MOUSE	3,20
Nucleobindin-1 OS=Mus musculus OX=	NUCB1_MOUSE	3,21
Fructose-bisphosphate aldolase A OS=	ALDOA_MOUSE	3,23
Glutathione S-transferase omega-1 O	GSTO1_MOUSE	3,23
Nidogen-1 OS=Mus musculus OX=100	NID1_MOUSE	3,24
Collagen alpha-2(IV) chain OS=Mus m	CO4A2_MOUSE	3,25
Cluster of 14-3-3 protein gamma OS=	1433G_MOUSE	3,25
Renin receptor OS=Mus musculus OX=	RENR_MOUSE	3,25
N-acetylgalactosamine-6-sulfatase OS	GALNS_MOUSE	3,25
Collagen alpha-2(I) chain OS=Mus mu	CO1A2_MOUSE	3,26
Isoform 2 of MICOS complex subunit M	MIC60_MOUSE	3,33
Metalloproteinase inhibitor 1 OS=Mus	TIMP1_MOUSE	3,35
Proteasome subunit beta type-1 OS=M	PSB1_MOUSE	3,38
Fibulin-5 OS=Mus musculus OX=10090	FBLN5_MOUSE	3,38
Elongation factor 1-gamma OS=Mus m	EF1G_MOUSE	3,38
Lipoprotein lipase OS=Mus musculus C	LIPL_MOUSE	3,39
Isoform 2 of Adenylate kinase 2, mitoc	KAD2_MOUSE	3,40
Integrin beta-1 OS=Mus musculus OX=	ITB1_MOUSE	3,43
Plasmalemma vesicle-associated prot	PLVAP_MOUSE	3,43
Cluster of Collagen alpha-1(XVIII) chai	COIA1_MOUSE	3,44
Palmitoyl-protein thioesterase 1 OS=M	PPT1_MOUSE	3,44
14-3-3 protein eta OS=Mus musculus (	1433F_MOUSE	3,46
Complement C1q subcomponent subu	C1QA_MOUSE	3,48
Testin-2 OS=Mus musculus OX=10090	TEST2_MOUSE	3,50
Platelet glycoprotein Ib beta chain OS=	GP1BB_MOUSE	3,50
Malate dehydrogenase, cytoplasmic O	MDHC_MOUSE	3,50
Isoform 2 of Sorcin OS=Mus musculus	SORCN_MOUSE	3,50
Protein SEC13 homolog OS=Mus musc	SEC13_MOUSE	3,50
Protein disulfide-isomerase A4 OS=Mu	PDIA4_MOUSE	3,50
Beta-1,3-N-acetylglucosaminyltransfe	LFNG_MOUSE	3,50
Platelet-activating factor acetylhydrola	PA1B2_MOUSE	3,50
26S proteasome non-ATPase regulato	PSD13_MOUSE	3,50
Cathepsin L1 OS=Mus musculus OX=10	CATL1_MOUSE	3,52
Nucleoside diphosphate kinase B OS=M	NDKB_MOUSE	3,52
Triosephosphate isomerase OS=Mus m	TPIS_MOUSE	3,55
Poly(rC)-binding protein 1 OS=Mus mu	PCBP1_MOUSE	3,56
Collagen alpha-1(I) chain OS=Mus mu	CO1A1_MOUSE	3,57
Proteasome subunit beta type-5 OS=M	PSB5_MOUSE	3,60
Proteasome subunit alpha type-3 OS=	PSA3_MOUSE	3,60

60S ribosomal protein L12 OS=Mus m	RL12_MOUSE	3,60
Biliverdin reductase A OS=Mus muscu	BIEA_MOUSE	3,60
Thioredoxin-like protein 1 OS=Mus mu	TXNL1_MOUSE	3,60
Glutathione S-transferase P 1 OS=Mus	GSTP1_MOUSE	3,63
Peroxiredoxin-2 OS=Mus musculus OX	PRDX2_MOUSE	3,64
Protein CYR61 OS=Mus musculus OX=	CYR61_MOUSE	3,64
Lysosomal alpha-mannosidase OS=Mu	MA2B1_MOUSE	3,65
Osteopontin OS=Mus musculus OX=10	OSTP_MOUSE	3,67
Rho GDP-dissociation inhibitor 2 OS=M	GDIR2_MOUSE	3,67
Follistatin-related protein 3 OS=Mus r	FSTL3_MOUSE	3,67
Fibrinogen alpha chain OS=Mus muscu	FIBA_MOUSE	3,68
Bone morphogenetic protein 1 OS=Mu	BMP1_MOUSE	3,75
Selenoprotein P OS=Mus musculus OX	SEPP1_MOUSE	3,75
14-3-3 protein epsilon OS=Mus muscu	1433E_MOUSE	3,77
Carbonic anhydrase 1 OS=Mus muscul	CAH1_MOUSE	3,81
Metalloproteinase inhibitor 2 OS=Mus	Q6PI17_MOUSE	3,85
Glucose-6-phosphate isomerase OS=M	G6PI_MOUSE	3,86
Proteasome subunit alpha type-1 OS=	PSA1_MOUSE	3,89
Cluster of Glutathione S-transferase M	GSTM1_MOUSE	3,92
Peroxiredoxin-1 OS=Mus musculus OX	PRDX1_MOUSE	3,92
Cluster of Phosphoglycerate mutase 1	PGAM1_MOUSE	3,94
Transgelin-2 OS=Mus musculus OX=10	TAGL2_MOUSE	3,98
Coatomer subunit beta' OS=Mus musc	COPB2_MOUSE	4,00
Isoform CNPI of 2',3'-cyclic-nucleotide	CN37_MOUSE	4,00
Actin-related protein 2/3 complex sub	ARPC5_MOUSE	4,00
Proteasome subunit alpha type-2 OS=	PSA2_MOUSE	4,00
Isoform VEGF-1 of Vascular endotheli	VEGFA_MOUSE	4,00
Enoyl-CoA delta isomerase 1, mitocho	ECI1_MOUSE	4,00
Apolipoprotein A-I OS=Mus musculus	APOA1_MOUSE	4,00
Ubiquitin-like modifier-activating enzy	UBA1_MOUSE	4,00
Vasodilator-stimulated phosphoprotei	VASP_MOUSE	4,00
Collagen alpha-2(VI) chain OS=Mus m	CO6A2_MOUSE	4,00
60S ribosomal protein L13a OS=Mus n	RL13A_MOUSE	4,00
Citrate synthase, mitochondrial OS=M	CISY_MOUSE	4,00
Ubiquitin-fold modifier-conjugating en	UFC1_MOUSE	4,00
Collectin-12 OS=Mus musculus OX=10	COL12_MOUSE	4,00
Cytochrome c oxidase subunit 4 isofor	COX41_MOUSE	4,00
Gamma-aminobutyric acid receptor-a	GBRAP_MOUSE	4,00
Inhibin beta B chain OS=Mus musculu	INHBB_MOUSE	4,00
Macrophage mannose receptor 1 OS=	MRC1_MOUSE	4,00
S-methyl-5'-thioadenosine phosphoryl	MTAP_MOUSE	4,00
UV excision repair protein RAD23 hom	RD23B_MOUSE	4,00
Isoform 2 of Neural cell adhesion mol	NCAM1_MOUSE	4,00
Calreticulin OS=Mus musculus OX=100	CALR_MOUSE	4,00
Matrix remodeling-associated protein	MXRA8_MOUSE	4,00
ATP synthase subunit gamma, mitoch	ATPG_MOUSE	4,00
SPARC-like protein 1 OS=Mus musculu	SPRL1_MOUSE	4,00
Obg-like ATPase 1 OS=Mus musculus	OLA1_MOUSE	4,00

26S proteasome non-ATPase regulato	PSDE_MOUSE	4,00
Cluster of Fibronectin OS=Mus muscul	A0A087WR50_MOUSE	4,01
F-actin-capping protein subunit alpha-	CAZA1_MOUSE	4,17
Ganglioside GM2 activator OS=Mus m	SAP3_MOUSE	4,22
Isoform 2 of Immunoglobulin heavy co	IGHM_MOUSE	4,25
Ribonuclease T2-A OS=Mus musculus	RNT2A_MOUSE	4,29
Connective tissue growth factor OS=M	CTGF_MOUSE	4,32
Protein disulfide-isomerase OS=Mus r	PDIA1_MOUSE	4,36
S-formylglutathione hydrolase OS=Mu	H3BKH6_MOUSE	4,40
Transaldolase OS=Mus musculus OX=1	TALDO_MOUSE	4,42
Carbonic anhydrase 2 OS=Mus muscul	CAH2_MOUSE	4,44
Cluster of Thrombospondin-1 OS=Mus	Q80YQ1_MOUSE	4,48
EMILIN-1 OS=Mus musculus OX=1009	EMIL1_MOUSE	4,50
Isoform IGF-IB of Insulin-like growth f	IGF1_MOUSE	4,50
Ear6 protein OS=Mus musculus OX=10	Q923L7_MOUSE	4,50
Germinal-center associated nuclear pr	GANP_MOUSE	4,50
14-3-3 protein zeta/delta OS=Mus mu	1433Z_MOUSE	4,58
Vitamin K-dependent protein S OS=Mu	PROS_MOUSE	4,60
40S ribosomal protein S5 (Fragment)	D3YYM6_MOUSE	4,60
N(4)-(beta-N-acetylglucosaminyl)-L-as	ASPG_MOUSE	4,67
Filamin-B OS=Mus musculus OX=1009	FLNB_MOUSE	4,68
Calcium-activated chloride channel reg	CA3A1_MOUSE	4,80
Proteasome subunit alpha type-5 OS=	PSA5_MOUSE	4,83
Dihydrolipoyllysine-residue succinyltra	ODO2_MOUSE	5,00
Integrin alpha-5 OS=Mus musculus OX	ITA5_MOUSE	5,00
Immunoglobulin superfamily member	IGSF8_MOUSE	5,00
Thioredoxin domain-containing protein	TXD12_MOUSE	5,00
Arginine-rich, mutated in early stage t	Q3TMX5_MOUSE	5,00
H-2 class I histocompatibility antigen,	G3UXW2_MOUSE	5,00
Heterogeneous nuclear ribonucleoprot	ROAA_MOUSE	5,00
Complement C1q tumor necrosis factc	C1QT5_MOUSE	5,00
Isoform 2 of Heterogeneous nuclear ri	HNRPD_MOUSE	5,00
Isoform 2 of Acyl-protein thioesterase	LYPA1_MOUSE	5,00
Extracellular sulfatase Sulf-2 OS=Mus	SULF2_MOUSE	5,00
Inorganic pyrophosphatase OS=Mus m	IPYR_MOUSE	5,00
Isoform 2 of Poly(rC)-binding protein 2	PCBP2_MOUSE	5,00
Isoamyl acetate-hydrolyzing esterase	IAH1_MOUSE	5,00
NPC intracellular cholesterol transport	NPC2_MOUSE	5,19
Proteasome subunit beta type-6 OS=M	PSB6_MOUSE	5,25
Isoform 2 of Sorbin and SH3 domain-c	SRBS2_MOUSE	5,25
Isoform 2 of Heterogeneous nuclear ri	ROA2_MOUSE	5,33
Annexin A1 OS=Mus musculus OX=100	ANXA1_MOUSE	5,36
Isoform 2 of Tenascin OS=Mus muscu	TENA_MOUSE	5,37
Cluster of Isoform 2 of Tropomyosin a	TPM3_MOUSE	5,40
Cluster of Tubulin beta-5 chain OS=Mu	TBB5_MOUSE	5,41
Beta-glucuronidase OS=Mus musculus	BGLR_MOUSE	5,48
Annexin A5 OS=Mus musculus OX=100	ANXA5_MOUSE	5,50
Isoform 2 of Proliferation-associated p	PA2G4_MOUSE	5,50

Ephrin-A1 OS=Mus musculus OX=1009	EFNA1_MOUSE	5,50
Acid ceramidase OS=Mus musculus O	ASAH1_MOUSE	5,60
26S proteasome non-ATPase regulato	PSMD8_MOUSE	5,67
40S ribosomal protein S4, X isoform O	RS4X_MOUSE	5,67
V-type proton ATPase subunit S1 OS=M	VAS1_MOUSE	5,70
Talin-1 OS=Mus musculus OX=10090 (	TLN1_MOUSE	5,73
F-actin-capping protein subunit alpha-	CAZA2_MOUSE	5,75
Serpin B6 OS=Mus musculus OX=1009	SPB6_MOUSE	5,87
Ferritin OS=Mus musculus OX=10090 (	Q9CPX4_MOUSE	5,88
Collagen alpha-1(V) chain OS=Mus mu	CO5A1_MOUSE	5,88
Malectin OS=Mus musculus OX=10090	MLEC_MOUSE	6,00
Isoform 2 of Calsyntenin-1 OS=Mus m	CSTN1_MOUSE	6,00
Calpain small subunit 1 OS=Mus musc	CPNS1_MOUSE	6,00
Ras GTPase-activating-like protein IQ	IQGA1_MOUSE	6,00
Hydroxyacyl-coenzyme A dehydrogena	HCDH_MOUSE	6,00
Decorin OS=Mus musculus OX=10090	PGS2_MOUSE	6,00
Spermidine synthase OS=Mus musculu	SPEE_MOUSE	6,00
Cluster of ADP-ribosylation factor 1 OS	ARF1_MOUSE	6,00
Dihydropteridine reductase OS=Mus m	DHPR_MOUSE	6,00
Isoform 2 of Stabilin-1 OS=Mus muscu	STAB1_MOUSE	6,00
Isoform 2 of Vascular endothelial grov	VEGFC_MOUSE	6,00
Elongation factor 1-beta OS=Mus mus	EF1B_MOUSE	6,00
Isoform 2 of Amyloid-like protein 2 OS	APLP2_MOUSE	6,00
LIM and SH3 domain protein 1 OS=Mu	LASP1_MOUSE	6,00
Serine protease 23 OS=Mus musculus	PRS23_MOUSE	6,00
Semaphorin-3A OS=Mus musculus OX	SEM3A_MOUSE	6,00
Creatine kinase B-type OS=Mus muscu	KCRB_MOUSE	6,00
Beta-1,4-galactosyltransferase 5 OS=M	B4GT5_MOUSE	6,00
Stromelysin-3 OS=Mus musculus OX=1	MMP11_MOUSE	6,00
Ferritin heavy chain OS=Mus musculus	FRIH_MOUSE	6,20
Bisphosphoglycerate mutase OS=Mus	PMGE_MOUSE	6,20
Isoform 2 of Platelet endothelial cell a	PECA1_MOUSE	6,25
Arylsulfatase A OS=Mus musculus OX=	ARSA_MOUSE	6,33
Ubiquitin-60S ribosomal protein L40 C	RL40_MOUSE	6,38
Collagen alpha-1(III) chain OS=Mus m	CO3A1_MOUSE	6,38
Moesin OS=Mus musculus OX=10090 (	MOES_MOUSE	6,40
Proteasome subunit alpha type-7 OS=	PSA7_MOUSE	6,40
6-phosphogluconolactonase OS=Mus m	6PGL_MOUSE	6,50
Deoxyribonuclease-2-alpha OS=Mus m	DNS2A_MOUSE	6,50
Growth factor receptor-bound protein	GRB2_MOUSE	6,50
Eukaryotic initiation factor 4A-I OS=M	IF4A1_MOUSE	6,50
Aspartate aminotransferase, mitochon	AATM_MOUSE	6,57
Vimentin OS=Mus musculus OX=1009(	VIME_MOUSE	6,63
Thrombospondin-2 OS=Mus musculus	TSP2_MOUSE	6,67
Dihydropyrimidinase-related protein 2	DPYL2_MOUSE	6,67
Complement C3 OS=Mus musculus OX	CO3_MOUSE	7,00
Receptor of activated protein C kinase	RACK1_MOUSE	7,00
Protein/nucleic acid deglycase DJ-1 OS	PARK7_MOUSE	7,00

Proteasome subunit beta type-2 OS=M	PSB2_MOUSE	7,00
ELAV-like protein 1 OS=Mus musculus	ELAV1_MOUSE	7,00
Actin-related protein 2/3 complex sub	ARPC4_MOUSE	7,00
Isoform 2 of Spectrin alpha chain, non	SPTN1_MOUSE	7,00
Mesothelin OS=Mus musculus OX=100	MSLN_MOUSE	7,00
Isoform 1 of Core histone macro-H2A.	H2AY_MOUSE	7,00
IST1 homolog OS=Mus musculus OX=1	IST1_MOUSE	7,00
Coatomer subunit delta OS=Mus musc	COPD_MOUSE	7,00
Inosine triphosphate pyrophosphatase	ITPA_MOUSE	7,00
Pyridoxal phosphate homeostasis prot	PLPHP_MOUSE	7,00
Cluster of Glyceraldehyde-3-phosphate	G3P_MOUSE	7,09
Peptidyl-prolyl cis-trans isomerase B (	PPIB_MOUSE	7,13
Cathepsin S OS=Mus musculus OX=10	CATS_MOUSE	7,15
Protein CREG1 OS=Mus musculus OX=	CREG1_MOUSE	7,25
Phosphoserine aminotransferase OS=I	SERC_MOUSE	7,25
Rab GDP dissociation inhibitor beta OS	GDIB_MOUSE	7,29
Collagen alpha-1(VI) chain OS=Mus m	CO6A1_MOUSE	7,40
Transgelin OS=Mus musculus OX=100	TAGL_MOUSE	7,50
L-lactate dehydrogenase B chain OS=M	LDHB_MOUSE	7,50
Transketolase OS=Mus musculus OX=1	TKT_MOUSE	7,60
von Willebrand factor OS=Mus muscu	VWF_MOUSE	7,93
Protein-glutamine gamma-glutamyltr	TGM2_MOUSE	8,00
Phosphatidylethanolamine-binding pro	PEBP1_MOUSE	8,00
Heme oxygenase 1 OS=Mus musculus	HMOX1_MOUSE	8,00
Proteasome subunit beta type-3 OS=M	PSB3_MOUSE	8,00
Neuronal pentraxin-1 OS=Mus muscul	NPTX1_MOUSE	8,00
Actin-related protein 2/3 complex sub	ARC1B_MOUSE	8,00
Electron transfer flavoprotein subunit	ETFA_MOUSE	8,00
Proteasome subunit beta type-8 OS=M	PSB8_MOUSE	8,00
Hypoxia up-regulated protein 1 OS=Mu	HYOU1_MOUSE	8,00
Cluster of Isoform 2 of Heterogeneous	HNRPF_MOUSE	8,00
Farnesyl pyrophosphate synthase OS=	FPPS_MOUSE	8,00
Myristoylated alanine-rich C-kinase su	MARCS_MOUSE	8,00
Isocitrate dehydrogenase [NADP], mit	IDHP_MOUSE	8,00
Purine nucleoside phosphorylase OS=M	PNPH_MOUSE	8,20
Peroxiredoxin-6 OS=Mus musculus OX	PRDX6_MOUSE	8,36
Isoform 2 of F-actin-capping protein s	CAPZB_MOUSE	8,40
Isoform Short of Collagen alpha-1(XI) (	COBA1_MOUSE	8,50
GTP-binding nuclear protein Ran OS=M	RAN_MOUSE	8,50
Collagen, type VI, alpha 3 OS=Mus mu	E9PWQ3_MOUSE	9,00
Isoform Short of Latent-transforming g	LTBP1_MOUSE	9,00
Proteasome subunit beta type-4 OS=M	PSB4_MOUSE	9,00
UMP-CMP kinase OS=Mus musculus O	KCY_MOUSE	9,00
40S ribosomal protein S8 OS=Mus mu	RS8_MOUSE	9,00
Electron transfer flavoprotein subunit	ETFB_MOUSE	9,00
Cathepsin O OS=Mus musculus OX=10	CATO_MOUSE	9,00
Olfactomedin-like protein 2B OS=Mus	OLM2B_MOUSE	9,00
Vascular endothelial growth factor rec	VGFR1_MOUSE	9,00

Isoform 2 of Multimerin-2 OS=Mus m	MMRN2_MOUSE	9,00
Isoform 2 of Septin-11 OS=Mus muscu	SEP11_MOUSE	9,00
Matrix metalloproteinase-19 OS=Mus	MMP19_MOUSE	9,00
Cluster of Elongation factor 1-alpha 1	EF1A1_MOUSE	9,13
Lysosomal acid lipase/cholesteryl este	LICH_MOUSE	9,50
Fascin OS=Mus musculus OX=10090 G	FSCN1_MOUSE	9,57
Inositol monophosphatase 1 OS=Mus	IMPA1_MOUSE	10,00
Superoxide dismutase [Mn], mitochon	SODM_MOUSE	10,00
Ras suppressor protein 1 OS=Mus mus	RSU1_MOUSE	10,00
Angiopoietin-related protein 2 OS=Mu	ANGL2_MOUSE	10,00
Destrin OS=Mus musculus OX=10090 (	DEST_MOUSE	10,00
Laminin subunit gamma-1 OS=Mus m	F8VQJ3_MOUSE	10,00
Ras-related protein Rab-7a OS=Mus m	RAB7A_MOUSE	10,00
Adenosylhomocysteinase OS=Mus mu	SAHH_MOUSE	10,00
Coronin-1C OS=Mus musculus OX=100	COR1C_MOUSE	10,00
Trifunctional enzyme subunit alpha, m	ECHA_MOUSE	10,00
Cell division control protein 42 homolo	CDC42_MOUSE	10,00
Mammalian ependymin-related prote	EPDR1_MOUSE	10,00
Ubiquitin-conjugating enzyme E2 K OS	UBE2K_MOUSE	10,00
Keratin, type I cytoskeletal 16 OS=Mus	K1C16_MOUSE	10,00
Peptidyl-prolyl cis-trans isomerase C C	PPIC_MOUSE	10,18
Rho GDP-dissociation inhibitor 1 OS=M	GDIR1_MOUSE	10,33
Cluster of Pyruvate kinase PKM OS=Mu	KPYM_MOUSE	10,46
NK13 OS=Mus musculus OX=10090 GN	O08804_MOUSE	10,67
Dihydrolipoyl dehydrogenase, mitocho	DLDH_MOUSE	11,00
Glutathione peroxidase 3 OS=Mus mu	GPX3_MOUSE	11,00
Protein-lysine 6-oxidase OS=Mus mus	LYOX_MOUSE	11,00
Ribosomal protein OS=Mus musculus	Q5XJF6_MOUSE	11,00
Proteasome subunit beta type-7 OS=M	PSB7_MOUSE	11,00
Troponin I, cardiac muscle OS=Mus m	TNNI3_MOUSE	11,00
Collagen alpha-1(XV) chain OS=Mus m	COFA1_MOUSE	11,00
Phosphatidylinositol transfer protein a	PIPNA_MOUSE	11,00
Isoform 2 of Cytosol aminopeptidase (	AMPL_MOUSE	11,00
Isoform 2 of Vacuolar protein sorting-	VPS29_MOUSE	11,00
Urokinase-type plasminogen activator	UROK_MOUSE	11,27
Putative phospholipase B-like 2 OS=M	PLBL2_MOUSE	11,33
Gamma-interferon-inducible lysosoma	GILT_MOUSE	11,50
Proteasome subunit alpha type-6 OS=	PSA6_MOUSE	11,67
C-C motif chemokine 2 OS=Mus muscu	CCL2_MOUSE	12,00
Cluster of Ras-related protein Rab-1B	RAB1B_MOUSE	12,00
WD repeat-containing protein 1 OS=M	WDR1_MOUSE	12,00
Isoform A3B of Troponin T, cardiac mu	TNNT2_MOUSE	12,00
Laminin subunit alpha-4 OS=Mus mus	LAMA4_MOUSE	12,00
Myoglobin OS=Mus musculus OX=100	MYG_MOUSE	12,00
Serine protease HTRA1 OS=Mus musc	HTRA1_MOUSE	12,00
Dihydropyrimidinase-related protein 3	E9PWE8_MOUSE	12,67
Proteasome subunit alpha type-4 OS=	PSA4_MOUSE	13,00
PDZ and LIM domain protein 5 OS=Mu	PDLI5_MOUSE	13,00

60S ribosomal protein L7 OS=Mus mu	RL7_MOUSE	13,00
40S ribosomal protein S3a OS=Mus m	RS3A_MOUSE	13,00
Aconitate hydratase, mitochondrial OS	ACON_MOUSE	13,00
Isoform 2 of Neutral alpha-glucosidas	GANAB_MOUSE	13,00
Microtubule-associated protein RP/EB	MARE1_MOUSE	13,00
Isoform PLEC-1I of Plectin OS=Mus mu	PLEC_MOUSE	14,00
Ras-related protein Rab-11B OS=Mus	RB11B_MOUSE	14,00
Prolow-density lipoprotein receptor-re	LRP1_MOUSE	14,00
Macrophage metalloelastase OS=Mus	MMP12_MOUSE	14,33
Adenylyl cyclase-associated protein 1 (	CAP1_MOUSE	14,67
Peroxidasin homolog OS=Mus musculu	PXDN_MOUSE	14,88
Translationally-controlled tumor prote	TCTP_MOUSE	15,00
Protein CTLA-2-alpha OS=Mus muscul	CTL2A_MOUSE	15,00
ATP synthase subunit O, mitochondria	ATPO_MOUSE	15,00
Eukaryotic translation initiation factor	IF5A1_MOUSE	15,00
Nidogen-2 OS=Mus musculus OX=100	NID2_MOUSE	15,00
Vesicular integral-membrane protein	LMAN2_MOUSE	16,00
Semaphorin-7A OS=Mus musculus OX	SEM7A_MOUSE	16,00
Glucosamine-6-phosphate isomerase	GNPI1_MOUSE	16,00
Laminin subunit beta-1 OS=Mus musc	LAMB1_MOUSE	16,00
Basement membrane-specific hepara	E9PZ16_MOUSE	16,71
Tropomodulin-3 OS=Mus musculus OX	TMOD3_MOUSE	17,00
60 kDa heat shock protein, mitochondr	CH60_MOUSE	17,00
40S ribosomal protein S3 OS=Mus mu	RS3_MOUSE	17,00
Actin-related protein 2/3 complex sub	ARPC3_MOUSE	18,00
Desmin OS=Mus musculus OX=10090	DESM_MOUSE	18,00
Protein FAM3C OS=Mus musculus OX=	FAM3C_MOUSE	19,00
Elongation factor 2 OS=Mus musculus	EF2_MOUSE	19,33
Titin OS=Mus musculus OX=10090 GN	TITIN_MOUSE	20,00
Actin-related protein 2/3 complex sub	ARPC2_MOUSE	20,50
ATP synthase subunit beta, mitochond	ATPB_MOUSE	20,57
Epididymis-specific alpha-mannosidas	MA2B2_MOUSE	21,00
Fibrillin-1 OS=Mus musculus OX=1009	FBN1_MOUSE	21,25
Glypican-4 OS=Mus musculus OX=100	GPC4_MOUSE	22,00
40S ribosomal protein S2 OS=Mus mu	RS2_MOUSE	23,00
Myosin regulatory light chain 2, ventri	MLRV_MOUSE	26,00
Cysteine and glycine-rich protein 1 OS	CSRP1_MOUSE	29,00
Ras-related C3 botulinum toxin substr	RAC1_MOUSE	29,00
Myosin light chain 3 OS=Mus musculu	MYL3_MOUSE	34,00
Cluster of Tropomyosin alpha-1 chain (	TPM1_MOUSE	59,00
ATP synthase subunit alpha, mitochon	ATPA_MOUSE	131,00
Myosin-6 OS=Mus musculus OX=1009	MYH6_MOUSE	Only in AngII
Angiopoietin-1 OS=Mus musculus OX=	ANGP1_MOUSE	Only in AngII
Cluster of Ras-related protein Rap-1b	RAP1B_MOUSE	Only in AngII
Cluster of Myosin-binding protein C, ca	E9Q9T8_MOUSE	Only in AngII
Creatine kinase M-type OS=Mus musc	KCRM_MOUSE	Only in AngII
Sepiapterin reductase OS=Mus muscu	SPRE_MOUSE	Only in AngII
Beta-mannosidase OS=Mus musculus	A0A0R4J092_MOUSE	Only in AngII

Delta(3,5)-Delta(2,4)-dienoyl-CoA ison	ECH1_MOUSE	Only in AngII
Pro-cathepsin H OS=Mus musculus OX	CATH_MOUSE	Only in AngII
60S ribosomal protein L18 OS=Mus m	RL18_MOUSE	Only in AngII
60S ribosomal protein L5 OS=Mus mu	RL5_MOUSE	Only in AngII
T-complex protein 1 subunit eta OS=M	TCPH_MOUSE	Only in AngII
3-ketoacyl-CoA thiolase, mitochondria	THIM_MOUSE	Only in AngII
Isoform Mt-VDAC1 of Voltage-depend	VDAC1_MOUSE	Only in AngII
Spectrin beta chain, non-erythrocytic 1	SPTB2_MOUSE	Only in AngII
60S ribosomal protein L6 OS=Mus mu	RL6_MOUSE	Only in AngII
Cytochrome c oxidase subunit 5A, mit	COX5A_MOUSE	Only in AngII
Mannosyl-oligosaccharide 1,2-alpha-m	MA1A1_MOUSE	Only in AngII
Isoform 2 of Disintegrin and metallopr	ADA15_MOUSE	Only in AngII
Isoform Cytoplasmic+peroxisomal of P	PRDX5_MOUSE	Only in AngII
Isoform 2 of Heterogeneous nuclear ri	HNRPU_MOUSE	Only in AngII
Isoform Short of H-2 class II histocomp	HG2A_MOUSE	Only in AngII
Tartrate-resistant acid phosphatase ty	PPA5_MOUSE	Only in AngII
Fibroblast growth factor 2 OS=Mus m	FGF2_MOUSE	Only in AngII
Peptidyl-prolyl cis-trans isomerase FK	FKB10_MOUSE	Only in AngII
BTB/POZ domain-containing protein K	KCD12_MOUSE	Only in AngII
NAD(P) transhydrogenase, mitochondr	NNTM_MOUSE	Only in AngII
Proprotein convertase subtilisin/kexin	PCSK9_MOUSE	Only in AngII
Proteasome activator complex subunit	PSME1_MOUSE	Only in AngII
60S ribosomal protein L14 OS=Mus m	RL14_MOUSE	Only in AngII
60S ribosomal protein L7a OS=Mus m	RL7A_MOUSE	Only in AngII
Isoform 2 of AP-2 complex subunit bet	AP2B1_MOUSE	Only in AngII
Metalloproteinase inhibitor 3 OS=Mus	TIMP3_MOUSE	Only in AngII
Cytoskeleton-associated protein 4 OS=	CKAP4_MOUSE	Only in AngII
Glutamate dehydrogenase 1, mitocho	DHE3_MOUSE	Only in AngII
Protein FAM49B OS=Mus musculus OX	FA49B_MOUSE	Only in AngII
Latent-transforming growth factor bet	LTBP2_MOUSE	Only in AngII
60S ribosomal protein L18a OS=Mus n	RL18A_MOUSE	Only in AngII
40S ribosomal protein S18 OS=Mus m	RS18_MOUSE	Only in AngII
Twinfilin-1 OS=Mus musculus OX=100	TWF1_MOUSE	Only in AngII
60S ribosomal protein L9 OS=Mus mu	RL9_MOUSE	Only in AngII
Cluster of Ubiquitin-conjugating enzyn	UB2V1_MOUSE	Only in AngII
ATP synthase subunit delta, mitochon	ATPD_MOUSE	Only in AngII
Clathrin heavy chain 1 OS=Mus muscu	CLH1_MOUSE	Only in AngII
Glutathione peroxidase 1 OS=Mus mu	GPX1_MOUSE	Only in AngII
Inhibin beta A chain OS=Mus musculu	INHBA_MOUSE	Only in AngII
Stromelysin-1 OS=Mus musculus OX=	MMP3_MOUSE	Only in AngII
Osteoclast-stimulating factor 1 OS=M	OSTF1_MOUSE	Only in AngII
Phosphatidylinositol transfer protein b	PIPNB_MOUSE	Only in AngII
60S ribosomal protein L13 OS=Mus m	RL13_MOUSE	Only in AngII
Acetyl-CoA acetyltransferase, mitocho	THIL_MOUSE	Only in AngII
Nuclease-sensitive element-binding p	YBOX1_MOUSE	Only in AngII
Synaptobrevin homolog YKT6 OS=Mus	YKT6_MOUSE	Only in AngII
AHNAK nucleoprotein (desmoyokin) O	E9Q616_MOUSE	Only in AngII
Isoform Smooth muscle of Myosin ligh	MYL6_MOUSE	Only in AngII

COP9 signalosome complex subunit 8	CSN8_MOUSE	Only in AngII
Tyrosine-protein kinase Mer OS=Mus r	MERTK_MOUSE	Only in AngII
40S ribosomal protein S7 OS=Mus mu	RS7_MOUSE	Only in AngII
T-complex protein 1 subunit zeta OS=I	TCPZ_MOUSE	Only in AngII
Isoform 2 of Matrilin-2 OS=Mus musc	MATN2_MOUSE	Only in AngII
[Protein ADP-ribosylarginine] hydrolas	ADPRH_MOUSE	Only in AngII
C-X-C motif chemokine 16 OS=Mus mu	CXL16_MOUSE	Only in AngII
Major prion protein OS=Mus musculus	PRIO_MOUSE	Only in AngII
Cluster of Isoform 2 of Microtubule-as	MAP4_MOUSE	Only in AngII
Fatty acid synthase OS=Mus musculus	FAS_MOUSE	Only in AngII
Hypoxanthine-guanine phosphoribosyl	HPRT_MOUSE	Only in AngII
Isopentenyl-diphosphate Delta-isomer	IDI1_MOUSE	Only in AngII
Integral membrane protein 2B OS=Mu	ITM2B_MOUSE	Only in AngII
Proteasome activator complex subunit	PSME2_MOUSE	Only in AngII
Cytochrome b-c1 complex subunit 2, m	QCR2_MOUSE	Only in AngII
Ubiquitin-conjugating enzyme E2 L3 O	UB2L3_MOUSE	Only in AngII
Isoform 2 of Thioredoxin reductase 1,	TRXR1_MOUSE	Only in AngII
Voltage-dependent anion-selective ch	VDAC2_MOUSE	Only in AngII
Cluster of ADP/ATP translocase 1 OS=	ADT1_MOUSE	Only in AngII
ATP synthase subunit d, mitochondrial	ATP5H_MOUSE	Only in AngII
Mannose-1-phosphate guanyltransfera	GMPPB_MOUSE	Only in AngII
Peroxiredoxin-4 OS=Mus musculus OX	PRDX4_MOUSE	Only in AngII
Cytochrome b-c1 complex subunit 1, m	QCR1_MOUSE	Only in AngII
Alpha-soluble NSF attachment protein	SNAA_MOUSE	Only in AngII
Syntaxin-7 OS=Mus musculus OX=100	STX7_MOUSE	Only in AngII
Fibulin-1 OS=Mus musculus OX=10090	FBLN1_MOUSE	Only in AngII
PDZ and LIM domain protein 7 OS=Mu	PDLI7_MOUSE	Only in AngII
Calcium-regulated heat stable protein	CHSP1_MOUSE	Only in AngII
Latexin OS=Mus musculus OX=10090 (	LXN_MOUSE	Only in AngII
26S proteasome non-ATPase regulato	PSD11_MOUSE	Only in AngII
T-complex protein 1 subunit beta OS=	TCPB_MOUSE	Only in AngII
Vesicle-associated membrane protein	VAPA_MOUSE	Only in AngII
Isoform 3 of Soluble calcium-activated	CANT1_MOUSE	Only in AngII
Isoform 2 of Adenylate kinase isoenzy	KAD1_MOUSE	Only in AngII
UDP-N-acetylhexosamine pyrophospho	UAP1L_MOUSE	Only in AngII
N(G),N(G)-dimethylarginine dimethyla	DDAH1_MOUSE	Only in AngII
Endothelial cell-selective adhesion mo	ESAM_MOUSE	Only in AngII
Lysosome-associated membrane glyco	LAMP1_MOUSE	Only in AngII
Profilin-1 OS=Mus musculus OX=1009	PROF1_MOUSE	Only in AngII
RNA-binding protein 3 OS=Mus muscu	RBM3_MOUSE	Only in AngII
Vacuolar protein sorting-associated pr	VPS35_MOUSE	Only in AngII
Catalase OS=Mus musculus OX=10090	CATA_MOUSE	Only in AngII
Xaa-Pro dipeptidase OS=Mus musculu	PEPD_MOUSE	Only in AngII
